# Bayesian reinforcement learning for navigation planning in unknown environments

**DOI:** 10.3389/frai.2024.1308031

**Published:** 2024-07-04

**Authors:** Mohammad Alali, Mahdi Imani

**Affiliations:** Department of Electrical and Computer Engineering, Northeastern University, Boston, MA, United States

**Keywords:** rescue operations, Markov decision process, reinforcement learning, Bayesian decision-making, navigation planning

## Abstract

This study focuses on a rescue mission problem, particularly enabling agents/robots to navigate efficiently in unknown environments. Technological advances, including manufacturing, sensing, and communication systems, have raised interest in using robots or drones for rescue operations. Effective rescue operations require quick identification of changes in the environment and/or locating the victims/injuries as soon as possible. Several techniques have been developed in recent years for autonomy in rescue missions, including motion planning, adaptive control, and more recently, reinforcement learning techniques. These techniques rely on full knowledge of the environment or the availability of simulators that can represent real environments during rescue operations. However, in practice, agents might have little or no information about the environment or the number or locations of injuries, preventing/limiting the application of most existing techniques. This study provides a probabilistic/Bayesian representation of the unknown environment, which jointly models the stochasticity in the agent's navigation and the environment uncertainty into a vector called the belief state. This belief state allows offline learning of the optimal Bayesian policy in an unknown environment without the need for any real data/interactions, which guarantees taking actions that are optimal given all available information. To address the large size of belief space, deep reinforcement learning is developed for computing an approximate Bayesian planning policy. The numerical experiments using different maze problems demonstrate the high performance of the proposed policy.

## 1 Introduction

Advances in robotics, sensing, manufacturing, and communication in recent years have raised interest in the use of autonomous agents instead of humans for rescue missions. Examples include using robots for time-sensitive and dangerous rescue missions, such as response to earthquakes, mass shootings, hurricanes, and warfare zones. The utmost factor in rescue operations is quick identification of changes in an environment or locating victims/injuries in need of critical care.

**Figure 1:**
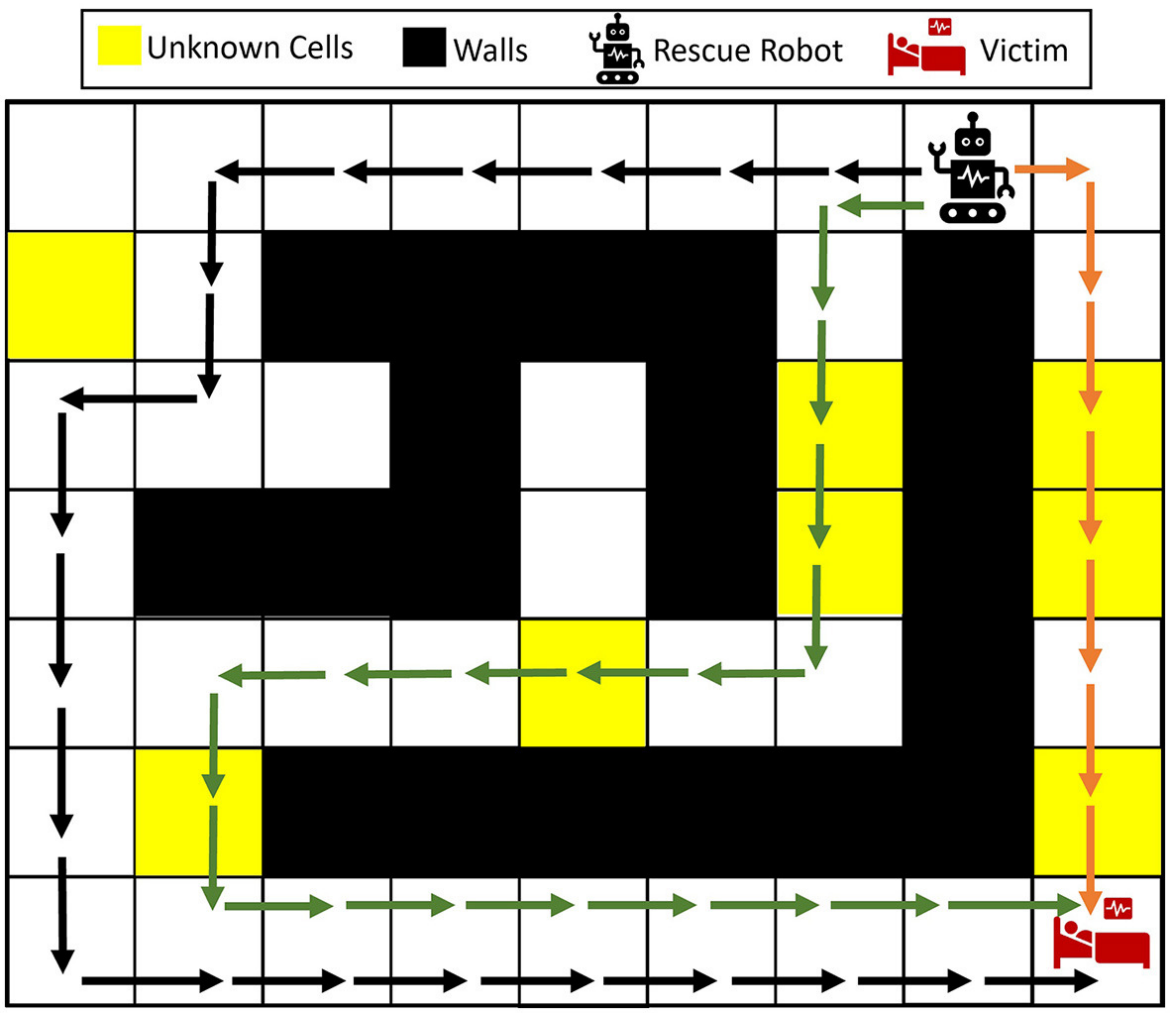
Illustrative example of the rescue operation problem in unknown environments. The rescue robot can take any of the black, green, and orange paths to get to the victim. Depending on the type of the unknown cells and whether each of the unknown cells is more probable to be wall, empty, or another victim/injury, the rescue robot should plan to take the best path.

A maze environment containing a single robot and a victim is shown in Figure 1. Let the yellow cells represent the unknown parts of the environment after the disaster, which could possibly be blocked with debris. Three possible navigation paths are shown in the maze. Given the unknown parts of the environment, should the agent take the right path, which is the closest to the victim? Wouldn't it be better to take the longer black path without any potential blockages (no yellow cells)? How can the robot change its decision as more information about the victim's condition and the environment appears?

Several techniques for achieving autonomy in rescue operations have been developed in recent years, with reinforcement learning (RL) being one of the prominent methods. In recent years, RL techniques have achieved remarkable success across various domains, including network security, biological applications, and robotics (Alali and Imani, 2023, 2024; Elguea-Aguinaco et al., 2023; Ravari et al., 2023; Alali et al., 2024; Asadi et al., 2024). For autonomy in rescue operations, various RL techniques have been developed for single-agent and multi-agent settings (Imanberdiyev et al., 2016; Zhang et al., 2018; Bøhn et al., 2019; Lin et al., 2019; Niroui et al., 2019; Sampedro et al., 2019; Ebrahimi et al., 2020; Hu et al., 2020; Wu et al., 2021). These RL techniques can be divided into model-based and simulation-based categories. The model-based RL approaches (Imanberdiyev et al., 2016; Pham et al., 2018; Ladosz et al., 2019; Sampedro et al., 2019; Xu et al., 2019) assume full knowledge (including potential changes/casualties) about the agent's environment during the rescue; the simulation-based RL techniques (Akcakoca et al., 2019; Wang et al., 2019; Blum et al., 2020; Hamid et al., 2021; Jagannath et al., 2021; Falcone and Putnam, 2022), on the other hand, rely on the availability of simulators to represent the environment during disaster response. However, the environment during the rescue operations is often unknown or partially known to the human and agent, and it is prudent for an agent to make decisions given the incomplete available information. For instance, in response to an earthquake, the number and location of victims/injuries and the extent of damage to the environment are often unknown at the early stages of rescue operations. This prevents the applicability of existing RL techniques in time-sensitive and unknown environments. It should also be noted that the RL techniques cannot be employed for learning a policy through real interactions with the unknown environment, as RL often requires thousands of interactions to learn to act in an unknown environment, which is impossible due to the time-sensitive nature of rescue operations.

Several techniques have been developed to combine Bayesian approaches with RL methods (Ghavamzadeh et al., 2015; Imani and Ghoreishi, 2022). Most of these approaches aim to address the sample efficiency of RL methods during the learning process (Ghavamzadeh et al., 2015; Imani et al., 2018; Kamthe and Deisenroth, 2018). Meanwhile, Bayesian approaches have been used to quantify the discrepancies between real-world environments and simulations, facilitating sim-to-real policy transfer (Feng et al., 2022; Rothfuss et al., 2024). Other Bayesian approaches have also been developed in multi-agent and human-AI teaming to learn the intentions and preferences of teammates using partial data (Lin et al., 2024; Ravari et al., 2024a,b; Zhang et al., 2024a,b). The most relevant class of approaches considering uncertainty in environments is Bayes-adaptive methods (Guez et al., 2012; Rigter et al., 2021; Zintgraf et al., 2021). These methods iteratively update the posterior distribution of the environment and simultaneously update the planning policy based on the latest interactions. However, these methods are applicable to domains with finite state spaces and fully unknown environments, where a huge number of interactions are needed to learn the distribution of the transition probabilities. These methods are also not applicable to partially known environments or domains with large and continuous state spaces and often perform poorly under limited available interactions.

Motion planning (Zhang et al., 2013; Perez-Imaz et al., 2016; Cabreira et al., 2019; de Almeida et al., 2019; Boulares and Barnawi, 2021) is another class of model-based approaches which aims to take advantage of the system model for offline planning. Examples of these methods are LQR/LQG methods and their non-linear variations (Richter and Roy, 2017; Kim et al., 2019, 2021; Bouman et al., 2020; Rosolia et al., 2022), which rely on the full or rich knowledge of the system model for planning or replanning, which prevents their applications in unknown environments. In this regard, active learning, model-predictive control, and online learning techniques have been developed for decision-making in unknown environments (Juang and Chang, 2011; Luo et al., 2014; Greatwood and Richards, 2019; Li et al., 2019; Chang et al., 2021). These methods mostly rely on a greedy and local view of the environment and perform poorly in complex realistic domains. Safety in navigation in unknown environments has also been studied extensively in the literature (Bajcsy et al., 2019; Krell et al., 2019; Tordesillas et al., 2019), which focuses on guaranteeing safety in domains with sensitive constraints.

This study develops a reinforcement learning Bayesian planning policy for rescue operations in unknown environments. We define a belief state, which keeps a joint probabilistic representation of all the possible models for the environment and agent movements. The belief state allows a Markov decision process (MDP) formulation of an agent in unknown and uncertain environments, allowing propagation of the entire uncertainty offline without the need for interaction with the real environment. We formulate the exact optimal Bayesian planning policy, which guarantees that an agent acts optimally given all available information. A Bayesian solution is introduced using a deep reinforcement learning technique, allowing offline learning of the policy over the whole belief space. We demonstrate that the proposed reinforcement learning Bayesian policy can be employed in real time for rescue missions in stationary and non-stationary environments as any additional information unfolds (i.e., without the need for learning or retraining). The effectiveness of the proposed method is demonstrated using comprehensive numerical experiments using different maze problems.

The article is organized as follows. In Section 2, the background of the Markov decision process is briefly described. In Section 3, the optimal Bayesian policy is formulated, and a solution based on deep reinforcement learning is introduced. Section 4 includes a discussion about the capabilities and complexity of the proposed method. Finally, Section 5 and Section 6 contain numerical examples and concluding remarks, respectively.

## 2 Background—A Markov decision process

A Markov decision process (MDP) can be defined by a 4-tuple $\langle S,A,T_{\theta },R_{\theta }\rangle$, where S is the *state space*, A is the *action space*, $T_{\theta }:S\times A\times S$ is the *unknown or partially known state transition probability function* such that $T_{\theta }\left(\text{s},\text{a},{\text{s}}^{\prime }\right)=P\left({\text{s}}^{\prime }|\text{s},\text{a},\theta \right)$ with the set of unknown parameters **θ** ∈ Θ, and $R_{\theta }:S\times A\times S\to \mathbb{R}$ is a bounded *reward function* with a real value outcome such that $R_{\theta }\left(\text{s},\text{a},{\text{s}}^{\prime }\right)$ encodes the reward earned when action **a** is taken in state **s** and the agent moves to state **s**′ in model **θ**. The reward function could be model-dependent in general form, meaning that similar transitions in different models of the environment might lead to different rewards.

If a given MDP parameterized by **θ** is a true environment, we could define a deterministic policy π:S→A as a mapping from states to actions. The expected discounted reward function at state s∈S after taking action a∈A and following policy π afterward is defined as follows:


(1) $$\begin{array}{l} \begin{array}{cc} q_{\theta }^{\pi }\left(\text{s},\text{a}\right)=𝔼[\overset{h}{\underset{t=0}{\sum }}\gamma^{t} & R_{\theta }\left({\text{s}}_{t},{\text{a}}_{t},{\text{s}}_{t+1}\right)|{\text{s}}_{0}=\text{s},{\text{a}}_{0}=\text{a},{\text{a}}_{1:\infty }\sim \pi ,\theta ]. \end{array} \end{array}$$

where γ is a discount factor. In the finite-horizon case, the discount factor is typically set to 1. In the infinite-horizon case (*h* = ∞), the discount factor γ ∈ [0, 1) is included to obtain a finite sum.

According to (1), the expected return under the optimal policy $\pi_{\theta }^{*}$ for the environment modeled by **θ** can be expressed as follows:


(2) $$\begin{array}{l} \begin{array}{c} q_{\theta }^{*}\left(\text{s},\text{a}\right)=𝔼[\overset{h}{\underset{t=0}{\sum }}\gamma^{t}R_{\theta }\left({\text{s}}_{t},{\text{a}}_{t},{\text{s}}_{t+1}\right)|{\text{s}}_{0}=\text{s},{\text{a}}_{0}=\text{a},{\text{a}}_{1:\infty }\sim \pi_{\theta }^{*},\theta ], \end{array} \end{array}$$

where $q_{\theta }^{*}\left(\text{s},\text{a}\right)$ indicates the expected discounted reward after executing action **a** in state **s** and following optimal policy $\pi_{\theta }^{*}$ afterward. An optimal model-specific policy $\pi_{\theta }^{*}$ attains the maximum expected return as $\pi_{\theta }^{*}\left(\text{s}\right)={\mathrm{argmax}}_{\text{a}\in A}q_{\theta }^{*}\left(\text{s},\text{a}\right)$, for s∈S.

If the true environment model, that is, θ^*^, was fully known to the agent, the optimal policy $\pi_{\theta^{*}}^{*}$ could be obtained using (2). However, the environment is often unknown in rescue operations, and the agent needs to make decisions given partial knowledge about the environment and/or the number or location of injuries.

## 3 Proposed Bayesian planning policy for decision-making in unknown environments

### 3.1 Probabilistic/Bayesian formulation of unknown and uncertain environments

**Figure 2:**
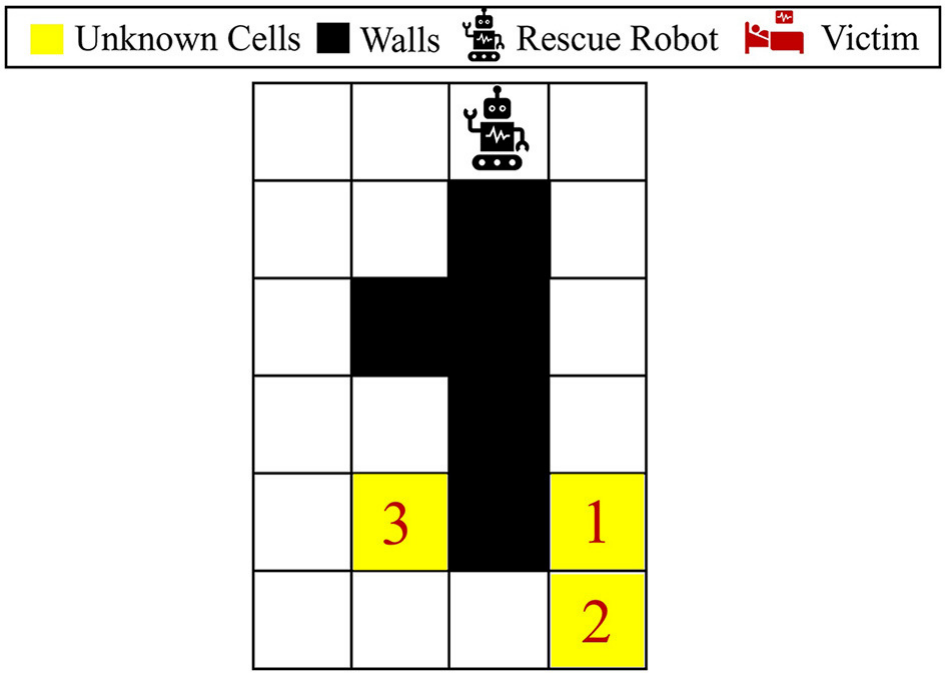
Visualization of the 6 × 4 maze problem. This maze has three unknown cells, where each could be either wall, empty, or victim/injury.

This section first describes the challenges of decision-making in an unknown environment, followed by the proposed framework to overcome these challenges effectively. Let us consider a maze problem as a simple example of navigation in rescue operations, where each cell in the maze could be one of the followings: wall “W”, empty “E”, or injury/victim “I”. Figure 2 represents an example of a maze problem, where the black and white colors indicate the wall and empty cells, respectively. The yellow cells are unknown parts of the environment, which each could potentially be a wall (i.e., blocked after a disaster), be empty, or contain a victim/injury. Let {*c*^1^, ..., *c*^*m*^} be *m* unknown cells in the environment, where *c*^*i*^ ∈ {*W, E, I*}. These unknown cells lead to 3^*m*^ different possible environment models (i.e., maze models) denoted by Θ = {**θ**^1^, ..., **θ**^3^*m*^^}, where **θ**^*j*^ = [**θ**^*j*^(1), ⋯  , **θ**^*j*^(*m*)] represents an MDP parametrized by **θ**^*j*^, and **θ**^*j*^(*l*) denotes the type of the *l*th unknown cell under the *j*th maze model. Note that the three unknown cells in Figure 2 lead to 3^3^ = 27 possible maze models, where the environments could potentially contain 0, 1, 2, or 3 injuries. In practice, the true environment θ^*^ is often hidden among all the possible models in Θ. For each **θ** ∈ Θ, the number and location of injuries and walls vary. Therefore, the optimal model-specific policies for these models [see Equation (2)] are different in general; thus, the policies obtained for different models **θ** ∈ Θ cannot be directly employed in unknown environments. It should be noted that the maze problem and its uncertain elements are examples of rescue missions, and the proposed method in this study could be applied to more general environments and environment uncertainties.

Let the initial knowledge about possible models for the true environment be represented as $\vartheta_{0}=\left[P\left(\theta^{*}=\theta^{1}\right),...,P\left(\theta^{*}=\theta^{3^{m}}\right)\right]$, where *P*(θ^*^ = **θ**^*j*^) shows the prior probability that the *j*th model is the true environment model and $\overset{3^{m}}{\underset{j=1}{\sum }}\vartheta_{0}\left(j\right)=1$. Let **a**_0:*k*−1_ = {**a**_0_, ..., **a**_*k*−1_} be the agent's actions and **s**_1:*k*_ = {**s**_1_, ..., **s**_*k*_} be the agent states until time step *k*. The posterior distribution of models can be expressed as follows:


(3) $$\begin{array}{l} \vartheta_{k}=\left[P\left(\theta^{*}=\theta^{1}|{\text{s}}_{1:k},{\text{a}}_{0:k-1}\right),\cdots \;,P\left(\theta^{*}=\theta^{3^{m}}|{\text{s}}_{1:k},{\text{a}}_{0:k-1}\right)\right], \end{array}$$

where ϑ_*k*_(*j*) indicates the posterior probability that model **θ**^*j*^ is the true model, and we also have $\overset{3^{m}}{\underset{j=1}{\sum }}\vartheta_{k}\left(j\right)=1$. Note that if no prior information about the models is available, a uniform (i.e., non-informative) distribution can be employed. If the independency of distribution of *m* unknown cells is assumed, the posterior probability could be defined over the *m* unknown cells as (Equation 4)


(4) $$\begin{array}{l} p_{k}=[P(c^{1}=W|s_{1:k},a_{0:k-1}),P(c^{1}=E|s_{1:k},a_{0:k-1}),\\ \;P(c^{1}=I|s_{1:k},a_{0:k-1}),\cdots ,P(c^{m}=W|s_{1:k},a_{0:k-1}),\\ \;P(c^{m}=E|s_{1:k},a_{0:k-1}),P(c^{m}=I|s_{1:k},a_{0:k-1})], \end{array}$$

which leads to the posterior distribution over all the possible models (i.e., ϑ_*k*_) as


(5) $$\begin{array}{l} \begin{array}{c} \vartheta_{k}\left(i\right)=\overset{m}{\underset{l=1}{\prod }}[1_{\theta^{i}\left(l\right)=W}p_{k}\left(3l-2\right)+1_{\theta^{i}\left(l\right)=E}p_{k}\left(3l-1\right)\\ +1_{\theta^{i}\left(l\right)=I}p_{k}\left(3l\right)], \end{array} \end{array}$$

for *i* = 1, ..., 3^*m*^ and 1_condition_ returns 1 if the condition is true, and 0 otherwise. Note that the rest of the study is derived for the general form of posterior in (3) without the independence assumption.

We define the *belief state* at time step *k* as the vector of joint agent's state (i.e., **s**_*k*_) and posterior probability of unknown models ϑ_*k*_:


(6) $$\begin{array}{l} {\text{b}}_{k}={\left[{\text{s}}_{k},\vartheta_{k}\right]}^{T}, \end{array}$$

where **b**_*k*_ is a vector of size $|{\text{s}}_{k}|+3^{m}$, and ${\text{b}}_{0}={\left[{\text{s}}_{0},\vartheta_{0}\right]}^{T}$ is the initial belief state. **s**_*k*_ is the agent state at time step *k* taking a value in S, and ϑ_*k*_ is a vector of size 3^*m*^, where each element takes continuous values between 0 and 1, and the sum of elements is 1. Thus, the space of belief state can be expressed as $B=\left\{S\times \Delta_{3^{m}}\right\}$, where $\Delta_{3^{m}}$ represents a simplex of size 3^*m*^.

### 3.2 MDP representation in belief space

In Section 3.1, we described that navigation in an unknown environment could be represented by an unknown MDP. Here, we show that the belief state defined in (6) allows representation of the navigation task in an unknown environment through a known MDP. The rationale behind this mapping is that, unlike in unknown MDPs, reinforcement learning techniques can be employed to find the optimal policy for a known MDP. In the following paragraphs, we first define the MDP in the belief space, then we represent all its elements, and finally, we formulate the reinforcement learning policy in this known MDP.

The MDP in the belief space can be expressed through $\langle B,A,\tilde{T},\tilde{R}\rangle$, where B is the belief state space, $\tilde{T}:B\times A\times B$ is a known transition probability in the belief space such that $\tilde{T}\left(\text{b},\text{a},{\text{b}}^{\prime }\right)=P\left({\text{b}}^{\prime }|\text{b},\text{a}\right)$ represents the transition from the belief state **b** to **b**′ if action **a** is taken. Note that [as proven in (7)] this transition is Markov and does not need the true model of the environment since the belief state contains the entire system uncertainty. Finally, the expected reward function in the belief state is represented by $\tilde{R}:B\times A\to \mathbb{R}$, where $\tilde{R}\left(\text{b},\text{a}\right)$ represents the expected immediate reward if action **a** is taken at belief state **b**.

Here, we provide proof that the belief transition is a Markov process. Let **b**_0_, **a**_0_, ..., **a**_*k*−1_, **b**_*k*_ be the sequence of actions and belief states up to time step *k*. If action **a**_*k*_ is taken at time step *k*, the probability of the next belief state can be expressed as follows:


(7) $$\begin{array}{l} P(b_{k+1}|a_{k},b_{k},...,b_{0},a_{0})=P(s_{k+1},\vartheta_{k+1}|a_{k},s_{k},\vartheta_{k},...,s_{0},\vartheta_{0},a_{0})\\ \;=P(s_{k+1}|a_{k},s_{k},\vartheta_{k},...,s_{0},\vartheta_{0},a_{0})\\ \;\times P(\vartheta_{k+1}|s_{k+1},a_{k},s_{k},\vartheta_{k},...,s_{0},\vartheta_{0},a_{0})\\ \;=P(s_{k+1}|a_{k},s_{k},\vartheta_{k})P(\vartheta_{k+1}|s_{k+1},a_{k},s_{k},\vartheta_{k})\\ \;=P(s_{k+1},\vartheta_{k+1}|a_{k},s_{k},\vartheta_{k})=P(b_{k+1}|a_{k},b_{k}), \end{array}$$

where the third line is written given the fact that ϑ_*k*_ includes the posterior distribution of models given all sequences of states and actions up to time step *k*. Therefore, the terms dropped in lines 2 and 3 of Equation (7) are already included in the posterior distribution ϑ_*k*_.

Given that **b** = [**s**, ϑ]^*T*^ is the current belief state, and **a** is the selected action at the current time, the next belief state can be one of the following |S| vectors:


(8) $$\begin{array}{l} {\text{b}}^{\prime }|\text{b},\text{a}\sim \{\begin{array}{ll} {\text{b}}_{1}^{\prime }={\left[{\text{s}}^{1},\vartheta_{1}^{\prime }\right]}^{T} & \text{w.p.}P\left({\text{b}}_{1}^{\prime }|\text{b},\text{a}\right)\\ {\text{b}}_{2}^{\prime }={\left[{\text{s}}^{2},\vartheta_{2}^{\prime }\right]}^{T} & \text{w.p.}P\left({\text{b}}_{2}^{\prime }|\text{b},\text{a}\right)\\ \vdots \\ {\text{b}}_{\left|S\right|}^{\prime }={\left[{\text{s}}^{\left|S\right|},\vartheta_{\left|S\right|}^{\prime }\right]}^{T} & \text{w.p.}P\left({\text{b}}_{\left|S\right|}^{\prime }|\text{b},\text{a}\right) \end{array}. \end{array}$$

The next belief state contains the next state and new posterior distribution of models. For instance, ${\text{b}}_{i}^{\prime }={\left[{\text{s}}^{i},\vartheta_{i}^{\prime }\right]}^{T}$ is one of S possible next belief states if the agent moves to state **s**^*i*^ after taking action **a** in state **s**. The posterior $\vartheta_{i}^{\prime }$ upon observing **s**^*i*^ can be computed as follows:


(9) $$\begin{array}{l} \begin{array}{c} \vartheta_{i}^{\prime }\left(j\right)=P\left(\theta^{*}=\theta^{j}|{\text{s}}^{\prime }={\text{s}}^{i},\text{a},\text{s},\vartheta \right)\\ =\frac{P\left({\text{s}}^{i}|\text{s},\text{a},\theta^{j}\right)\vartheta \left(j\right)}{\sum_{l=1}^{3^{m}}P\left({\text{s}}^{i}|\text{s},\text{a},\theta^{l}\right)\vartheta \left(l\right)}, \end{array} \end{array}$$

for $j=1,...,3^{m},i=1,...,|S|$.

The probability for all |S| possible next belief state transitions denoted in (9) can be computed according to the Markovian properties of the belief transition in (7). In particular, the probability that the *i*th belief state ${\text{b}}_{i}^{\prime }={\left[{\text{s}}^{i},\vartheta_{i}^{\prime }\right]}^{T}$ is observed upon taking action **a** in belief state **b** = [**s**, ϑ]^*T*^ can be expressed as follows:


(10) $$\begin{array}{l} \begin{array}{c} P\left({\text{b}}_{k+1}={\text{b}}_{i}^{\prime }|{\text{b}}_{k}=\text{b},{\text{a}}_{k}=\text{a}\right)\\ =P\left({\text{s}}_{k+1}={\text{s}}^{i},\vartheta_{k+1}=\vartheta_{i}^{\prime }|{\text{b}}_{k}=\text{b},{\text{a}}_{k}=\text{a}\right)\\ =P\left({\text{s}}_{k+1}={\text{s}}^{i}|{\text{s}}_{k}=\text{s},\vartheta_{k}=\vartheta ,{\text{a}}_{k}=\text{a}\right)\\ \times P\left(\vartheta_{k+1}=\vartheta_{i}^{\prime }|{\text{s}}_{k+1}={\text{s}}^{i},{\text{s}}_{k}=\text{s},\vartheta_{k}=\vartheta ,{\text{a}}_{k}=\text{a}\right)\\ =P\left({\text{s}}_{k+1}={\text{s}}^{i}|{\text{s}}_{k}=\text{s},\vartheta_{k}=\vartheta ,{\text{a}}_{k}=\text{a}\right). \end{array} \end{array}$$

The last line of (10) is obtained given that ϑ_*k*+1_ can only take a single value $\vartheta_{i}^{\prime }$ [computed in (9)] with probability 1.

The expected reward function $\tilde{R}\left(\text{b},\text{a}\right)$ in the belief space can be expressed in terms of the reward function of the true environment as follows:


(11) $$\begin{array}{l} \begin{array}{c} \tilde{R}\left(\text{b}={\left[\text{s},\vartheta \right]}^{T},\text{a}\right)=\underset{{\text{b}}^{\prime }\in \left\{{\text{b}}_{1}^{\prime },...,{\text{b}}_{\left|S\right|}^{\prime }\right\}}{\sum }P\left({\text{b}}^{\prime }={\left[{\text{s}}^{\prime },\vartheta^{\prime }\right]}^{T}|\text{b},\text{a}\right)E_{\theta |\vartheta^{\prime }}\left[R_{\theta }\left(\text{s},\text{a},{\text{s}}^{\prime }\right)\right]\\ =\overset{\left|S\right|}{\underset{i=1}{\sum }}P\left({\text{b}}_{i}^{\prime }={\left[{\text{s}}^{i},\vartheta_{i}^{\prime }\right]}^{T}|\text{b},\text{a}\right)\overset{3^{m}}{\underset{l=1}{\sum }}\vartheta_{i}^{\prime }\left(l\right)R_{\theta^{l}}\left(\text{s},\text{a},{\text{s}}^{i}\right), \end{array} \end{array}$$

where $R_{\theta^{l}}\left(\text{s},\text{a},{\text{s}}^{\prime }\right)$ represents the improvement in the rescue operation after taking action **a** and moving from state **s** to **s**′ in model **θ**^*l*^. It can be seen that the reward function in (11) depends on the posterior distribution of models and the uncertainty in the agent state transitions.

Aside from the ability to incorporate any arbitrary reward functions defined for the true environment, the proposed belief formulation allows actively exploring/learning the unknown parts of the environment. For instance, robots/drones might be deployed in rescue operations to quickly identify road closures or other environmental casualties. Depending on the application, the uncertainty at specific/targeted locations or the entire environment might be needed. The immediate gain in uncertainty reduction at belief state **b** = [**s**, ϑ]^*T*^ after taking action **a** can be expressed as follows:


(12) $$\begin{array}{l} \begin{array}{c} \tilde{R}\left(\text{b}={\left[\text{s},\vartheta \right]}^{T},\text{a}\right)=-{𝔼}_{{\text{b}}^{\prime }={\left[{\text{s}}^{\prime },\vartheta^{\prime }\right]}^{T}|\text{b},\text{a}}\left[H\left(\vartheta^{\prime }\right)-H\left(\vartheta \right)\right]\\ =-\overset{\left|S\right|}{\underset{i=1}{\sum }}P\left({\text{b}}_{i}^{\prime }|\text{b},\text{a}\right)\left[H\left(\vartheta_{i}^{\prime }\right)-H\left(\vartheta \right)\right]\\ =\overset{\left|S\right|}{\underset{i=1}{\sum }}P\left({\text{b}}_{i}^{\prime }|\text{b},\text{a}\right)\overset{3^{m}}{\underset{l=1}{\sum }}\left[\vartheta_{i}^{\prime }\left(l\right)\log\vartheta_{i}^{\prime }\left(l\right)-\vartheta \left(l\right)\log\vartheta \left(l\right)\right], \end{array} \end{array}$$

where *H*(ϑ) denotes the remaining entropy (i.e., uncertainty) in the environment model represented by the posterior probability ϑ. Larger positive reward values correspond to more reduction of entropy/uncertainty upon moving to belief state **b**′. The entropy takes its lowest value 0 when representing the case where a single model has posterior probability 1 and others 0. Therefore, this reward function helps agents toward taking actions that provide the highest information about unknown parts of the environment, which is crucial in rescue operations. Note that depending on our application, a more general form of the reward function can be employed for learning the navigation policy.

### 3.3 Deep reinforcement learning Bayesian planning policy

The MDP defined in the belief space is fully known as it considers the posterior of all the possible environment models. We define μ:B→A as a deterministic policy, which associates an action to each sample in the belief space. The optimal policy in the belief space can be formulated as follows:


(13) $$\begin{array}{l} \mu^{*}\left(\text{b}\right)=\underset{\mu }{\mathrm{argmax}}𝔼\left[\overset{\infty }{\underset{t=0}{\sum }}\gamma^{t}\tilde{R}\left({\text{b}}_{t},{\text{a}}_{t}\right)|{\text{b}}_{0}=\text{b},{\text{a}}_{0:\infty }\sim \mu \right], \end{array}$$

for any b∈B; where the maximization is over all possible policies in the belief space. The expectation in (13) is with respect to the stochasticity in the belief space denoted in (8), which includes the uncertainty in the state transition and the posterior of environment models reflected in the belief states. The optimal Bayesian policy, μ^*^, yields optimality given all available information reflected in the belief state (i.e., the agent and model uncertainty). Finding the exact solution for the optimization problem in (13) is not possible due to the large size of the belief space. In the following paragraphs, we provide an approximate solution for finding the optimal Bayesian policy in (13) using a deep reinforcement learning approach.

This study employs the belief transition in (8) as a simulator to generate offline trajectories required for training a deep RL agent. These trajectories are belief transitions that propagate the agent states and the environment uncertainty, thus, do not require interaction with the real environment. Given the discrete nature of action space, we employ the deep Q-network (DQN) method (Mnih et al., 2015) for learning the Bayesian policy in (13). This approach aims to approximate the following expected discounted reward function defined over the belief space:


(14) $$\begin{array}{l} Q^{*}\left(\text{b},\text{a}\right)=𝔼\left[\overset{\infty }{\underset{t=0}{\sum }}\gamma^{t}\tilde{R}\left({\text{b}}_{t},{\text{a}}_{t}\right)|{\text{b}}_{0}=\text{b},{\text{a}}_{0}=\text{a},{\text{a}}_{1:\infty }\sim \mu^{*}\right], \end{array}$$

for any b∈B and a∈A.

DQN approximates the Q-function in (14) using two feed-forward deep neural networks, called Q-network and target-network, represented by *Q*_**w**_ and $Q_{{\text{w}}^{-}}$, respectively. These two neural networks share the same structure; the Q-network's input is the belief state, and its outputs are $Q_{\text{w}}\left(\text{b},{\text{a}}^{1}\right),...,Q_{\text{w}}\left(\text{b},{\text{a}}^{|A|}\right)$, each associated with an action. The initial weights for both Q-network and target-network are set randomly.

For training of the neural networks, a replay memory D of fixed size is considered. This memory is filled and replaced by repeated episodes of belief states governed by actions generated from the epsilon-greedy policy. Each episode starts from an initial belief state ${\text{b}}_{0}={\left[{\text{s}}_{0},\vartheta_{0}\right]}^{T}$, which, if unknown, can be selected randomly from the belief space, that is, ${\text{b}}_{0}\in B$. At step *t* of the episode, an action can be selected according to the epsilon-greedy policy defined using the Q-network *Q*_**w**_ as follows:


(15) $$\begin{array}{l} {\text{a}}_{t}\sim \{\begin{array}{ll} {\mathrm{argmax}}_{\text{a}\in A}Q_{\text{w}}\left({\text{b}}_{t},\text{a}\right) & \text{w.p. }1-\epsilon \\ \text{random}\left\{{\text{a}}^{1},...,{\text{a}}^{|A|}\right\} & \text{w.p.}\epsilon \end{array}, \end{array}$$

where 0 ≤ ϵ ≤ 1 is the epsilon-greedy policy rate, which controls the level of exploration during the learning process.

Upon generating a fixed number of steps, the Q-network *Q*_**w**_ should be updated according to a minibatch set of experiences selected from the replay memory D. Letting


(16) $$\begin{array}{l} Z={\left\{\left({\tilde{\text{b}}}_{n},{\tilde{\text{a}}}_{n},{\tilde{\text{b}}}_{n+1},{\tilde{r}}_{n+1}\right)\right\}}_{n=1}^{N_{\text{batch}}}\sim D, \end{array}$$

be selected as a minibatch set, the target values for updating the Q-network can be computed as follows:


(17) $$\begin{array}{l} y_{n}={\tilde{r}}_{n+1}+\gamma \underset{\text{a}\in A}{\max}Q_{{\text{w}}^{-}}\left({\tilde{\text{b}}}_{n+1},\text{a}\right), \end{array}$$

for *n* = 1, ..., *N*_batch_, where the target-network $Q_{{\text{w}}^{-}}$ is used for computation of the target values. Using these target values, the Q-network weights, **w**, can be updated as follows:


(18) $$\begin{array}{l} \text{w}=\text{w}-\alpha \nabla_{\text{w}}\left[\overset{N_{\text{batch}}}{\underset{n=1}{\sum }}{\left(y_{n}-Q_{\text{w}}\left({\tilde{\text{b}}}_{n},{\tilde{\text{a}}}_{n}\right)\right)}^{2}\right], \end{array}$$

where α is the learning rate, and the mean squared error is used for the loss function in the weights update. The optimization in (18) can be carried out using a stochastic gradient optimization approach such as Adam (Kingma and Ba, 2015). Upon updating **w**, the weights of the target-network, **w**^−^, should also be updated using the soft update:


(19) $$\begin{array}{l} {\text{w}}^{-}=\left(1-\tau \right){\text{w}}^{-}+\tau \text{w}, \end{array}$$

where τ is a hyperparameter.

It should be noted that the trajectories used in the DQN method are acquired offline through the belief transition in (8). The training can be stopped when performance improvement becomes negligible, or a pre-specified performance is achieved. Upon termination of the offline training, the Q-network approximates the optimal Bayesian policy as $\mu^{*}\left(\text{b}\right)\approx {\mathrm{argmax}}_{\text{a}\in A}Q_{\text{w}}\left(\text{b},\text{a}\right)$. This Bayesian policy prescribes an action for any given belief state b∈B; thus, it can be employed in real time during the execution as the new belief state is calculated according to the real interactions with the environment. The major computations of the proposed policy are during the offline process; during the execution, the belief state needs to be tracked/updated, and the learned policy reflected in the trained Q-network is applied according to the latest belief.

## 4 Proposed Bayesian policy's complexity analysis and capabilities

In this section, we briefly describe the key differences between the proposed method and some of the well-known techniques for rescue operations. Then, we discuss the advantage and capabilities of the proposed method as well as the computational complexity for real-time implementation.

Let $q_{\theta }^{*}\left(\text{s},\text{a}\right)$ be the optimal expected return for model **θ** defined in (2). These values can be obtained offline using dynamic programming or reinforcement learning approaches tuned for any model **θ** ∈ Θ. It should be noted that the model-specific Q-values are defined over the original state space and not the belief space. Assuming **s**_*k*_ is the current state of the agent and ϑ_*k*_ is the posterior distribution of the environment models at time step *k*, the proposed optimal Bayesian policy formulated in (13) and approximated using the Q-network through (15)-(19) can be expressed in the belief space as follows:


(20) $$\begin{array}{l} \begin{array}{c} {\text{a}}_{k}=\underset{\text{a}\in A}{\mathrm{argmax}}Q^{*}\left({\text{b}}_{k},\text{a}\right)\approx \underset{\text{a}\in A}{\mathrm{argmax}}Q_{\text{w}}\left(\left[s_{k},\vartheta_{k}\right],\text{a}\right). \end{array} \end{array}$$

Another well-known approach commonly used for learning in unknown environments is the maximum aposteriori (MAP) navigation policy, which relies on the underlying model-specific policy with the highest posterior probability. This policy can be expressed as follows:


(21) $$\begin{array}{l} {\text{a}}_{k}=\underset{\text{a}\in A}{\mathrm{argmax}}q_{\theta_{k}^{\text{MAP}}}^{*}\left({\text{s}}_{k},\text{a}\right), \end{array}$$

where $\theta_{k}^{\text{MAP}}={\mathrm{argmax}}_{\theta^{i};i=1,...,3^{m}}\vartheta_{k}\left(i\right)$.

In addition, *active learning* approaches (and their variations) (Silver et al., 2012; Kaplan and Friston, 2018; Choudhury and Srinivasa, 2020; Taylor et al., 2021; Rckin et al., 2022, 2023) are widely used techniques for decision-making in unknown environments. As noted in their names, these methods aim to actively lookahead and evaluate the options. A one-step procedure can be expressed as follows:


(22) $$\begin{array}{l} {\text{a}}_{k}=\underset{\text{a}\in A}{\mathrm{argmax}}{𝔼}_{\vartheta_{k}}\left[q_{\theta }^{*}\left({\text{s}}_{k},\text{a}\right)\right]=\underset{\text{a}\in A}{\mathrm{argmax}}\overset{3^{m}}{\underset{i=1}{\sum }}\vartheta_{k}\left(i\right)q_{\theta^{i}}^{*}\left({\text{s}}_{k},\text{a}\right). \end{array}$$

Comparing policies in Equations (20–22), one can see that the primary computation of all the methods is done in an offline process. During the online execution, all methods require updating the belief state (or the posterior probability of models), followed by using the learned/computed Q-values obtained during the offline process. The MAP policy in Equation (21) relies on a single model (i.e., a model with the highest posterior probability). Thus, if several models yield the maximum posterior probability or the model with the highest posterior is not definitively distinguished from others, the decisions made by the MAP policy become unreliable. Unlike the MAP policy, the active learning approach in Equation (22) accounts for the posterior of all models for decision-making. This policy becomes more efficient in domains where the posterior is peaked over a single model. If the posterior distribution is uniform over the models, the active learning policy looks like averaging according to various models' Q-function. The main difference between active learning and the proposed method is the incapability of active learning methods to change the posterior of the models (i.e., acting to enhance modeling information that can lead to better rewards). The active learning decisions might keep the posterior distribution unchanged over time, meaning that all models (including the wrong models) contribute similarly in making decisions over time. By contrast, the proposed Bayesian policy in Equation (20) learns the policy over the state and posterior of models, meaning that the action selection optimally influences the agent state and the posterior of models in achieving the highest accumulated rewards. This can be seen as taking actions that lead to moving to belief states under which better navigation performance can be achieved.

Aside from the efficiency of the proposed Bayesian policy described above, another advantage of the proposed policy is the generality of learning. The generality of learning refers to the fact that the proposed policy could be employed for a wide range of objectives. As described in Equations (11, 12), the reward could be defined for locating victims in the environment, quick identification of the unknown parts of the environment (i.e., changing the posterior distribution of models) or any other reward functions that can be expressed using the belief state. However, the active learning and MAP policies in Equations (21, 22) can only consider the objectives (i.e., reward functions) that are defined according to the original state space (i.e., not the posterior of models). These capabilities are investigated and discussed in the next section through various numerical experiments.

Scalability could be a limitation of our proposed method. The size of the posterior distribution in Equation (3) grows exponentially as the number of unknown cells increases in the environment, and this leads to an increase in the size of the belief space. This increases the computational complexity of the proposed policy, making it intractable for domains with uncertainty represented in continuous (or infinite-dimensional) spaces. Note that the scalability of active learning and MAP policies also increases with the uncertainty in the environment models. Our future research will investigate approaches to scale the proposed Bayesian policy to large environments with possibly large and infinite number of environment models.

## 5 Numerical experiments

In this section, the performance of the proposed Bayesian policy is investigated using different maze problems with the following two objectives: (1) locating the victims/injuries in unknown environments as fast as possible; (2) exploring an unknown maze environment as quickly as possible, modeled through entropy reduction. Values of all the parameters used in our numerical experiments are presented as follows: number of hidden layers 3, number of neurons in hidden layers 128, α = 5 × 10^−4^, $|D|=10^{5}$, *N*_batch_ = 64, γ = 0.95, ϵ = 0.1, τ = 10^−3^, and the update frequency for the Q-network is considered to be 4. Note that all the experiments in this section are repeated for 1,000 trials, and the average results along the 95% confidence bounds are displayed in all the figures.

### 5.1 Locating injuries

#### 5.1.1 6 × 4 maze problem

For our first set of experiments, we consider the 6 × 4 maze shown in Figure 2. This maze consists of 5 walls and the agent can be in any of the other 19 cells at each step. Furthermore, the 3 unknown cells indicated by yellow lead to 3^3^ = 27 possible maze models. For the actions, the agent at each step can select right, left, down, or up. We also consider some stochasticity in the environment as the agent moves to the anticipated direction with probability of 0.8, or it will move to either of the perpendicular directions with probability of 0.1. Each of the unknown cells could contain an injury, be empty, or blocked by a wall. To guide the agent to track three potential injuries, we define three new auxiliary variables as η_*k*_ = {η_1_, η_2_, η_3_}, where each variable turns to 0 if an agent moves to the corresponding unknown states and locates an injury. Note that these auxiliary variables are needed to track multiple objectives in the environment. Therefore, the state space in this case contains the location of the agent (i.e., one of 19 possible locations in the maze) and the auxiliary variables. The belief space size in this case is $B=\left\{\left\{1,...,19\right\}\times {\left\{0,1\right\}}^{3}\times \Delta_{27}\right\}$. The reward function in the belief state is modeled using (11), with $R_{\theta }\left(\text{s},\text{a},{\text{s}}^{\prime }\right)=1$ if upon moving to **s**′ a new injury is located according to the maze model parameterized by **θ**, and $R_{\theta }\left(\text{s},\text{a},{\text{s}}^{\prime }\right)=0$ otherwise.

Here, we assume that the agent can identify empty cells and cells with injury if it moves to those states. Therefore, the transition probability for model **θ** required for the belief state transition in Equations (8, 9) can be expressed as follows:


(23) $$\begin{array}{l} P\left({\text{s}}^{i}|\text{s},\text{a},\theta \right)=\{\begin{array}{ll} p_{\text{s}{\text{s}}^{\prime }}^{\text{a},\theta }1_{t\left({\text{s}}^{i}\right)=\theta \left(l\right)} & \text{if }{\text{s}}^{i}={\text{s}}_{u}^{l}\\ p_{\text{s}{\text{s}}^{\prime }}^{\text{a},\theta } & \text{if }{\text{s}}^{i}\notin S_{u} \end{array}, \end{array}$$

where $S_{u}$ contains the unknown cells, ${\text{s}}_{\text{u}}^{l}$ is the *l*th unknown cell, and $p_{\text{s}{\text{s}}^{\prime }}^{\text{a},\theta }$ is 0.8 if **s**′ is the neighboring cell to **s** at the direction indicated by action **a** in the environment model **θ** and 0.1 if it is in cross perpendicular neighborhood. Note that *t*(**s**^*i*^) indicates the type of state **s**^*i*^; that is, *W*, *E* or *I*. In addition, $1_{t\left({\text{s}}^{i}\right)=\theta \left(l\right)}$ is 1 if the type of state **s**^*i*^ and unknown cell **θ**(*l*) is the same and zero otherwise. Using the defined *P*(**s**^*i*^ ∣ **s**, **a**, **θ**) and Equation (9), the posterior update in our problem can be explicitly formulated. According to Equation (5), we consider the independency assumption for the unknown cells, represented through the initial distribution $p_{0}=\left[p_{0}^{1},p_{0}^{2},p_{0}^{3}\right]$, where $p_{0}^{i}=\left[P\left(c^{i}=W\right),P\left(c^{i}=E\right),P\left(c^{i}=I\right)\right]$ consists of the prior probability of the *i*th unknown cell.

*ReLu* is used as an activation function between each layer of our neural networks. Furthermore, a maximum of 50 steps is considered for testing our proposed planning policy; however, a larger horizon of 250 steps is used for training purposes to account for the discounted rewards in the final steps. In addition, the proposed Bayesian planning policy is trained over 5,000 episodes in each case.

**Figure 3:**
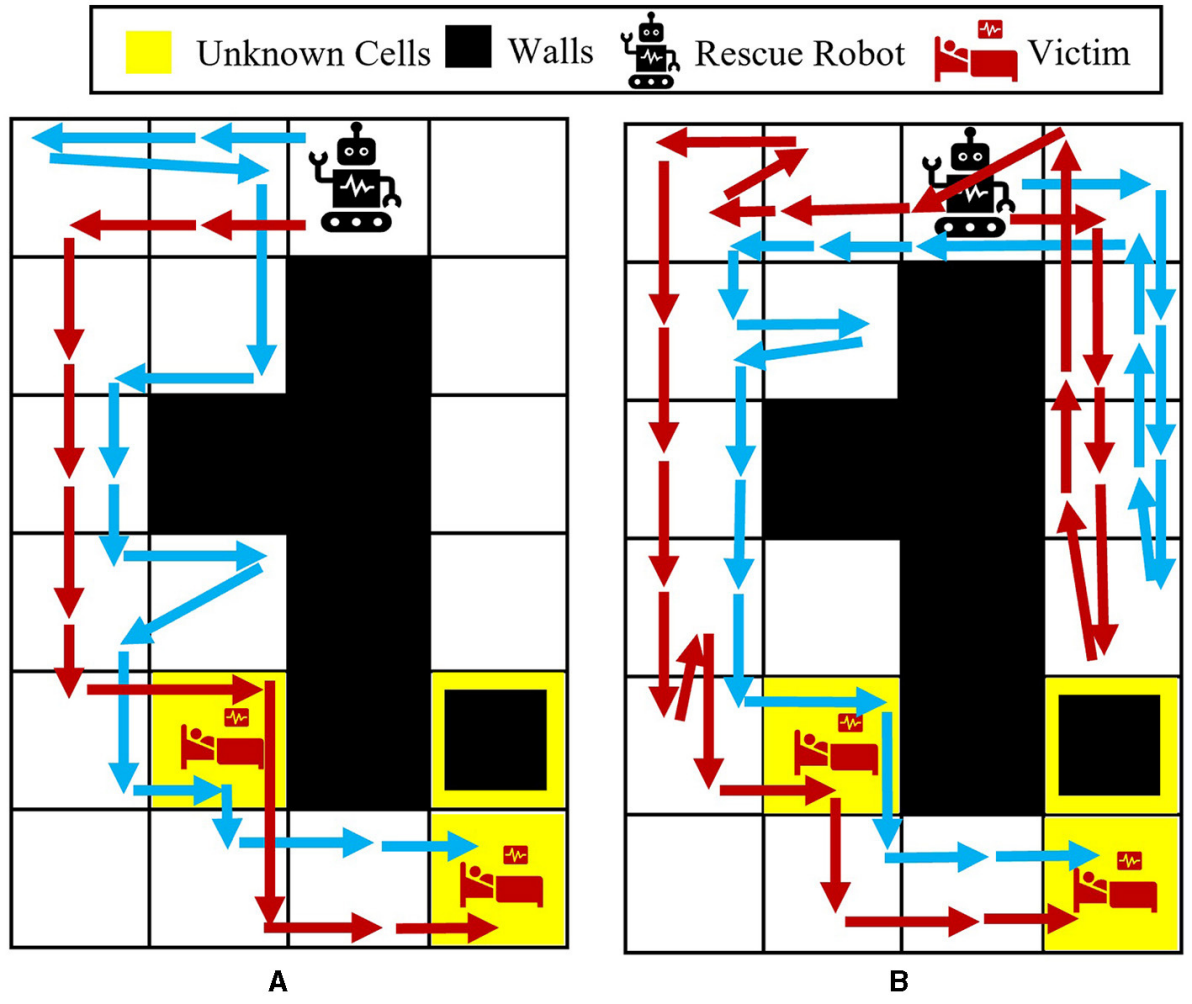
Agent movement trajectories under the proposed Bayesian policy in the 6 × 4 maze; each of the blue and red arrows corresponds to one independent movement trajectory, and the difference in the trajectories is due to the movement stochasticity in the environment. The trajectories are recorded using the proposed policy under two different initial distributions: **(A)** $p_{0}^{1}=\left[\frac{2}{3},\frac{1}{6},\frac{1}{6}\right],p_{0}^{2}=p_{0}^{3}=\left[\frac{1}{3},\frac{1}{3},\frac{1}{3}\right]$, **(B)** $p_{0}^{i}=\left[\frac{1}{3},\frac{1}{3},\frac{1}{3}\right]$ for *i* = 1, 2, 3.

To better show how different prior probabilities can affect the agent's decisions, we visualized the agent's movements in the 6 × 4 maze problem using two cases in Figure 3. The prior probabilities for all unknown cells are set to be equally distributed between wall, empty, and injury (i.e., $p_{0}^{i}=\left[\frac{1}{3},\frac{1}{3},\frac{1}{3}\right]$ for *i* = 1, 2, 3), except for the first unknown cell in the left maze, which is set to $p_{0}^{1}=\left[\frac{2}{3},\frac{1}{6},\frac{1}{6}\right]$. The true environment is assumed to include a wall in the first unknown cell and injuries in the other two unknown cells. The paths selected by the agent under the proposed Bayesian policy are indicated in each maze in Figure 3. One can see that the agent moves from left in Figure 3A since it has prior knowledge that the first unknown cell is likely to be a wall. However, in Figure 3B, the agent selects the right path since it predicts that this path leads to the quickest rescue operation given the equal prior probabilities for the unknown cells. Once the agent encounters the wall in the first unknown cell, the Bayesian policy guides it to go back and reach out to other potential injuries in the environment as quickly as possible. Note that the agent's movements are also uncertain, which can be seen as the difference between the two trajectories shown in each maze in Figure 3.

**Figure 4:**
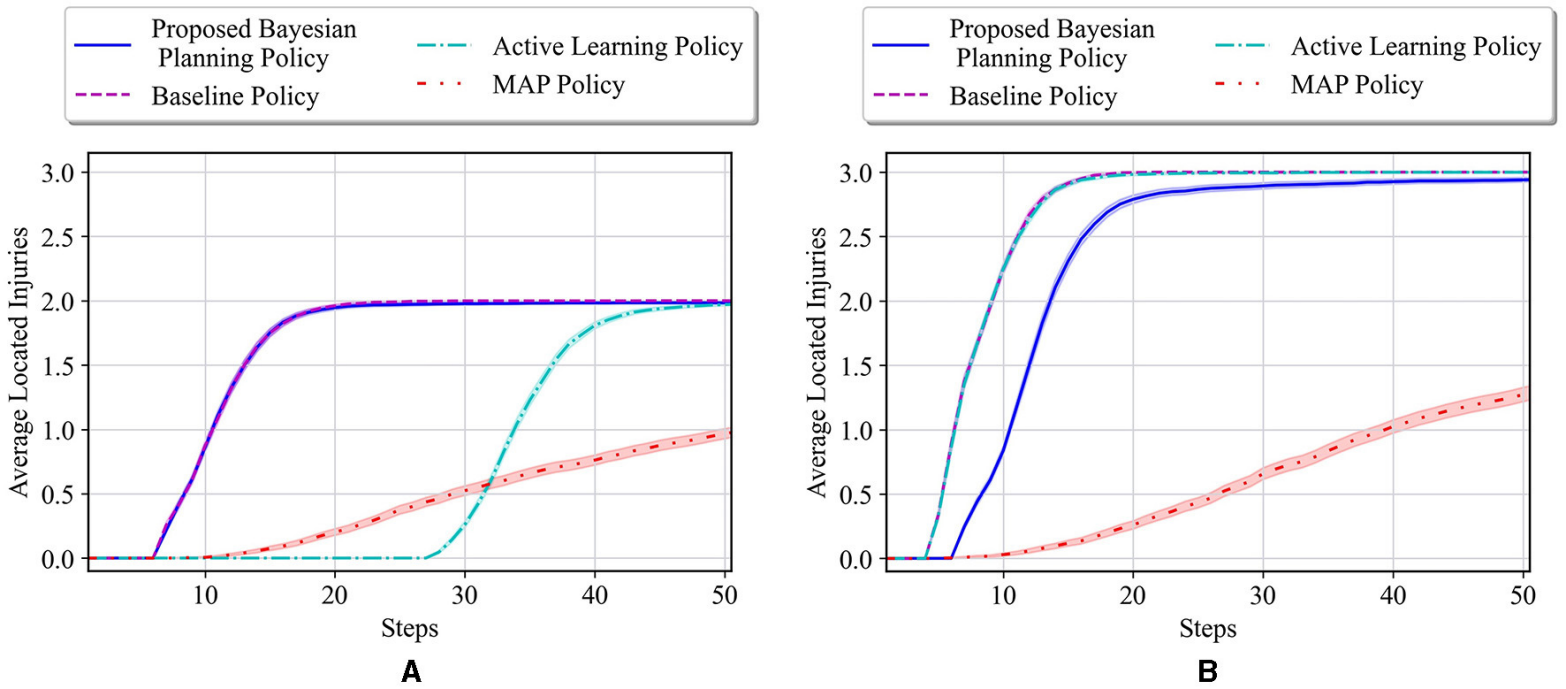
**(A)** Performance comparison in the 6 × 4 maze with the true environment θ^*^ = [*W, I, I*] and initial probabilities of unknown cells as $p_{0}^{1}=\left[\frac{2}{3},\frac{1}{6},\frac{1}{6}\right],p_{0}^{2}=p_{0}^{3}=\left[\frac{1}{3},\frac{1}{3},\frac{1}{3}\right]$. **(B)** Performance comparison in the 6 × 4 maze with the true environment θ^*^ = [*I, I, I*] and initial probabilities of unknown cells as $p_{0}^{1}=\left[\frac{2}{3},\frac{1}{6},\frac{1}{6}\right],p_{0}^{2}=p_{0}^{3}=\left[\frac{1}{3},\frac{1}{3},\frac{1}{3}\right]$.

The average results for the proposed policy are compared with three approaches; baseline approach, which is the reinforcement learning (i.e., Q-learning) solution when the true model of the environment is known, and MAP and active learning policies formulated in Equations (21, 22). The baseline solution is the best achievable solution if no uncertainty in the environment exists; thus, it is used to assess how uncertainty in the environment model can deviate the solutions of various policies from the solution in the known environment. Figure 4A shows the average located injuries and their confidence bounds using different navigation policies for 1,000 trials and over the first 50 steps for the maze environment shown in Figure 3A. As can be seen in Figure 4A, the proposed policy has a superior performance compared to the active learning and MAP policies as it matches the performance of the baseline policy in all the steps, and after only 20 steps it can find the two injuries in the environment successfully. Active learning policy is the next best policy in this case, and it reaches maximum performance after 50 steps. Notice that although active learning gets to all the injuries after 50 steps, it still has a very low performance in the first 30 steps, which is not desirable especially in situations where we should get to the injuries in a timely manner. In addition, we can see that the MAP policy has a better performance than active learning in the first 32 steps; however, active learning performance improves significantly after that and the MAP policy becomes the worst policy since it shows poor performance even after 50 steps.

In the second test environment, similar prior probabilities are used for our experiments (i.e., $p_{0}^{1}=\left[\frac{2}{3},\frac{1}{6},\frac{1}{6}\right],p_{0}^{2}=p_{0}^{3}=\left[\frac{1}{3},\frac{1}{3},\frac{1}{3}\right]$), while in the true environment, all the unknown cells are considered to be injuries. The obtained test results for this environment are shown in Figure 4B. In this figure, we can see that the active learning and baseline policies have the same performance, and our method has the second best performance. This is because in this test case all the unknown cells are injuries, whereas the prior in our case has been set to have a higher probability for wall for the first unknown cell. Regardless of setting a faulty prior for our method, after only 20 steps, our method shows a high performance in comparison with the baseline, and the performance keeps increasing to almost the same as the baseline as the steps increase. Moreover, we can see that the MAP policy again performs poorly as to other methods.

**Figure 5:**
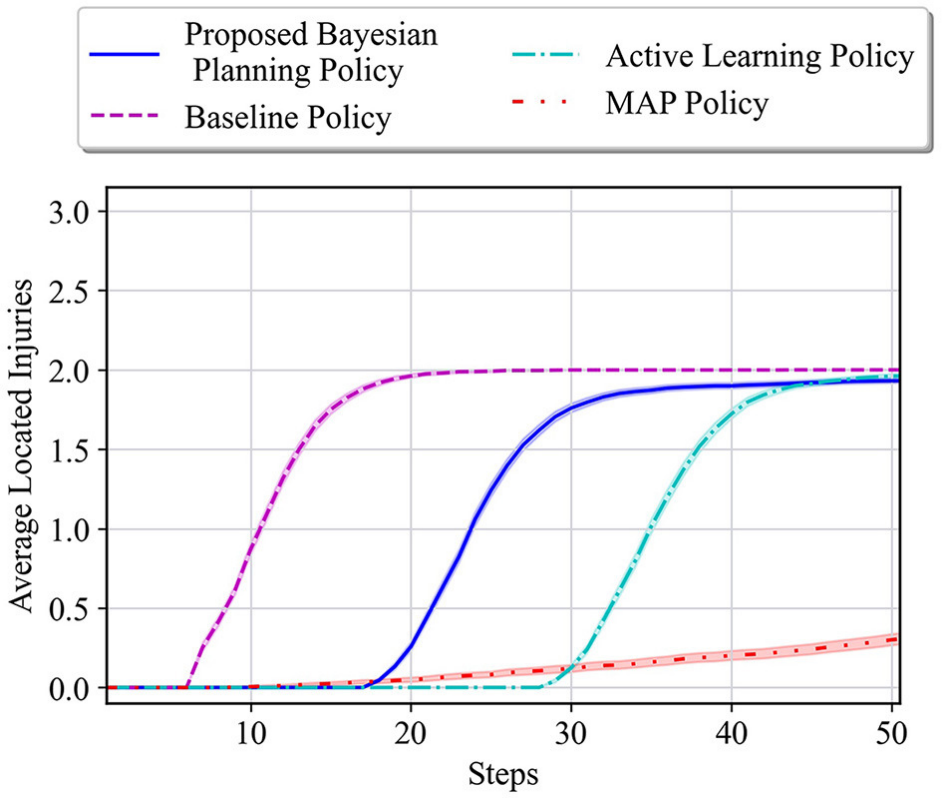
Performance comparison in the 6 × 4 maze with the true environment θ^*^ = [*W, I, I*] and initial probabilities of unknown cells as $p_{0}^{i}=\left[\frac{1}{3},\frac{1}{3},\frac{1}{3}\right]$ for *i* = 1, 2, 3.

Figure 5 shows the average results for the maze shown in Figure 3B. All the unknown cells have the same initial probability of being a wall, empty, or injury (i.e., $p_{0}^{i}=\left[\frac{1}{3},\frac{1}{3},\frac{1}{3}\right]$ for *i* = 1, 2, 3). One can see that our proposed policy has a better performance than active learning and MAP policy as it reaches a performance close to the baseline in only 30 steps. Active learning also reaches a good performance after about 40 steps; however, it has much lower performance than our approach specifically between steps 18 and 40. Finally, similar to the other previous cases, it can be observed that the MAP policy has the worst performance in comparison with others.

**Figure 6:**
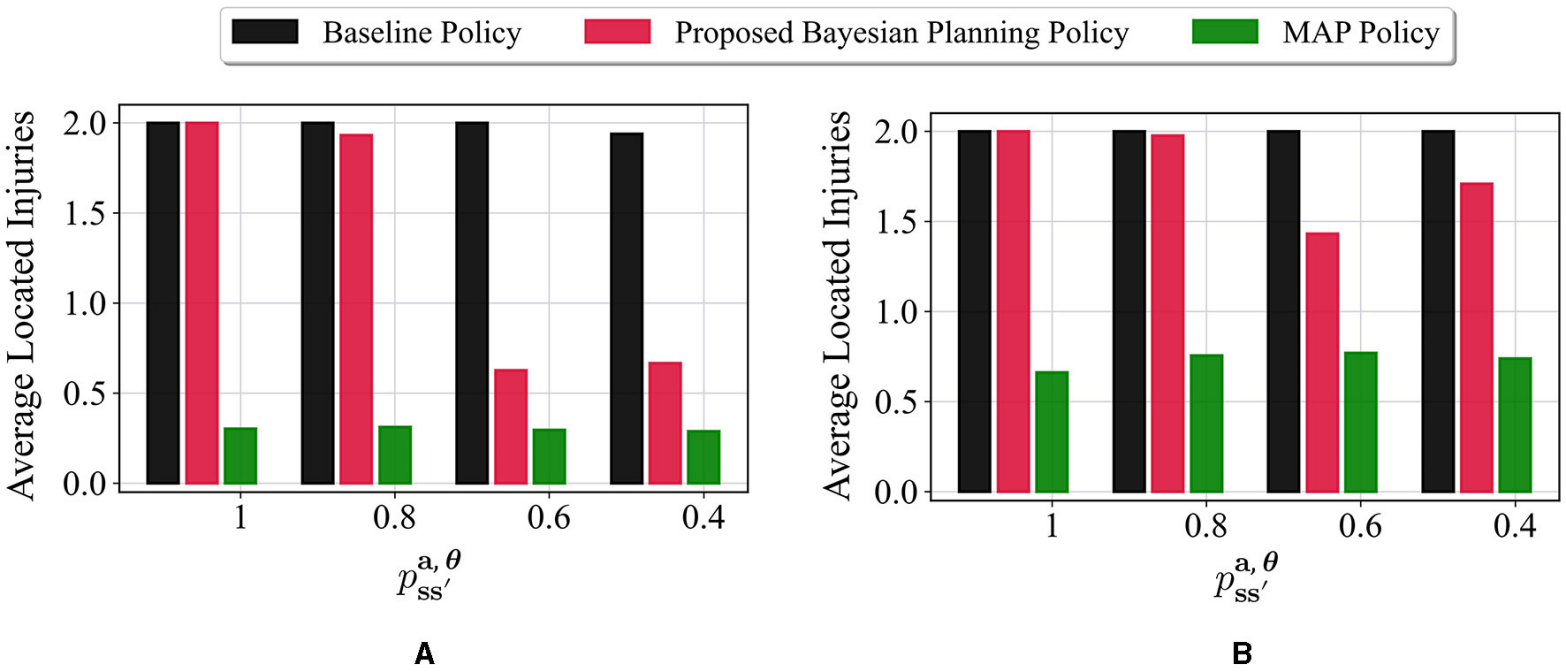
Impact of movement stochasticity ($p_{\text{s}{\text{s}}^{\prime }}^{\text{a},\theta }$) on the performance of different policies in the 6 × 4 maze with the true environment θ^*^ = [*W, I, I*] and initial probabilities of unknown cells as $p_{0}^{i}=\left[\frac{1}{3},\frac{1}{3},\frac{1}{3}\right]$ for *i* = 1, 2, 3. Higher values for $p_{\text{s}{\text{s}}^{\prime }}^{\text{a},\theta }$ correspond to more deterministic movements whereas lower values refer to more stochastic movements. The performance of different policies is shown in two timesteps: **(A)** Timestep 50 and **(B)** Timestep 100.

In the next set of experiments, the impact of different values for the parameter $p_{\text{s}{\text{s}}^{\prime }}^{\text{a},\theta }$ in Equation (23) on the performance of our proposed method is investigated. As described before, $p_{\text{s}{\text{s}}^{\prime }}^{\text{a},\theta }$ refers to the movement stochasticity in the maze; a higher value of $p_{\text{s}{\text{s}}^{\prime }}^{\text{a},\theta }$ means that the movement is more deterministic and the agent moves in the desired direction, and a lower value denotes that the agent movement is more stochastic and it is more likely to end up in one of the perpendicular directions. Four values of 1, 0.8, 0.6, and 0.4 are considered for $p_{\text{s}{\text{s}}^{\prime }}^{\text{a},\theta }$. These values are tested on the environment of Figure 3B, with all the unknown cells having the following prior probabilities: $p_{0}^{i}=\left[\frac{1}{3},\frac{1}{3},\frac{1}{3}\right]$ for *i* = 1, 2, 3. The average performance of the baseline method, the proposed method, and the MAP policy in terms of average located injuries are shown in Figure 6. Figure 6A represents the performance of different policies at timestep 50. It can be seen that the best performance of the proposed policy is achieved at $p_{\text{s}{\text{s}}^{\prime }}^{\text{a},\theta }=1$, where it has performed similarly to the baseline. The second best performance for the proposed policy occurs under the value $p_{\text{s}{\text{s}}^{\prime }}^{\text{a},\theta }=0.8$, where our policy has slightly smaller performance than the baseline policy. The performance of our method under the values 0.6 and 0.4 is lower and almost similar in both cases since both values represent environments with high movement stochasticity. Moreover, it can be seen that the worst performance is achieved by the MAP in all the cases. Furthermore, Figure 6B shows the performance after 100 timesteps. In this case, our method's performance under the values of 0.8, 0.6, and 0.4 gets much closer to the baseline policy, whereas the MAP policy still performs poorly. This clearly demonstrates the robustness of our proposed method under different stochasticity levels in the environment.

**Figure 7:**
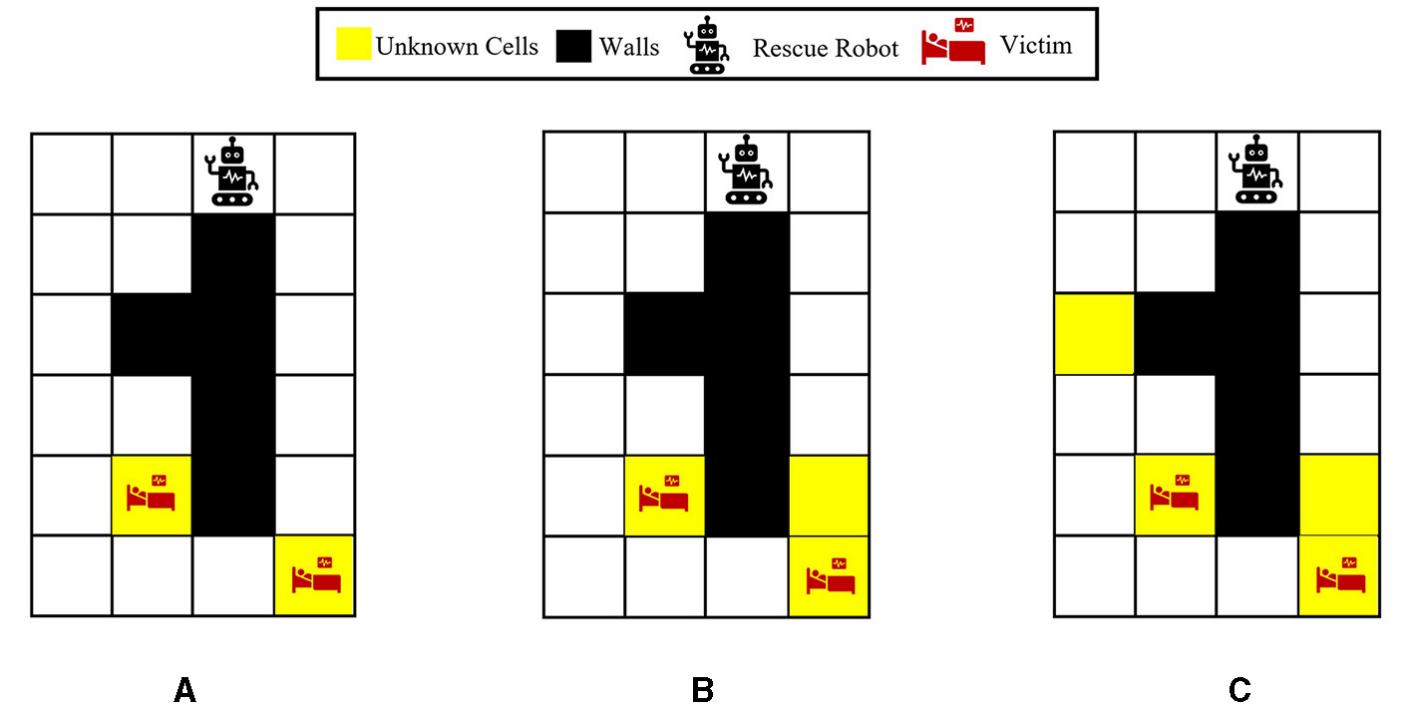
Visualization of the 6 × 4 grid problem with different numbers of unknown cells: **(A)** Two unknown cells. **(B)** Three unknown cells. **(C)** Four unknown cells. For a more fair comparison, as shown in all the grids, two of the unknown cells are considered to be victims/injuries in the true underlying environment (θ^*^) when performing the tests, and the rest of the unknown cells in **(B, C)** could be wall or empty.

For further analysis, the impact of different unknown cells is studied in the next set of experiments. We consider three variations of the 6 × 4 maze problem. Figures 7A–C represent the environments with two, three, and four unknown cells, respectively. A uniform prior probability is used for all the unknown cells during training, that is, $\left[\frac{1}{3},\frac{1}{3},\frac{1}{3}\right]$. Furthermore, for a fair comparison of these environments during the test, two of the unknown cells are assumed to contain victims/injuries as demonstrated in Figure 7, and the other unknown cells in environments (Figures 7B, C) can be either wall or empty. The average number of located injuries achieved in all the environments at timesteps 10, 20, and 30 using different policies is reported in Table 1. The performance of our proposed Bayesian policy is indicated by bold numbers. This table shows that at each timestep, the performance of our method decreases as the number of unknown cells (and the complexity of the maze) increases. Furthermore, one can see that the performance of our policy in all the environments and at all the timesteps surpasses other policies' performance, indicating the effectiveness of our approach. This indicates that our method is capable of reasoning about additional uncertainty in the environments to still make effective decisions.

**Table 1:** Average number of located injuries by different policies for locating two injuries in the 6 × 4 maze with different numbers of unknown cells.

**Timestep 10**			
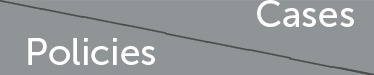	**Two unknown cells**	**Three Unknown Cells**	**Four unknown cells**
Proposed Policy	**0.957** **±0.017**	**0.72** **±0.053**	**0.25** **±0.03**
Active Learning	0.003 ± 0.001	0.514 ± 0.036	0.005 ± 0.001
MAP	0.016 ± 0.008	0.009 ± 0.006	0.009 ± 0.006
**Timestep 20**			
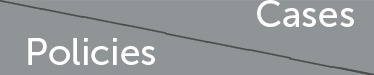	**Two unknown cells**	**Three unknown cells**	**Four unknown cells**
Proposed Policy	**1.2** **±0.03**	**0.961** **±0.06**	**0.89** **±0.056**
Active Learning	0.005 ± 0.001	0.761 ± 0.053	0.104 ± 0.025
MAP	0.137 ± 0.083	0.018 ± 0.008	0.073 ± 0.018
**Timestep 30**			
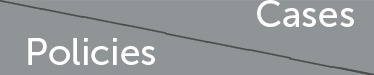	**Two unknown cells**	**Three unknown cells**	**Four unknown cells**
Proposed Policy	**1.395** **±0.035**	**1.347** **±0.055**	**1.191** **±0.055**
Active Learning	0.008 ± 0.002	1.005 ± 0.055	0.247 ± 0.04
MAP	0.301 ± 0.033	0.207 ± 0.029	0.17 ± 0.027

The bold numbers refer to the highest average number of located injuries among all the policies. As can be seen these bold numbers are achieved by the proposed policy in all the scenarios.

#### 5.1.2 4 × 4 maze problem

**Figure 8:**
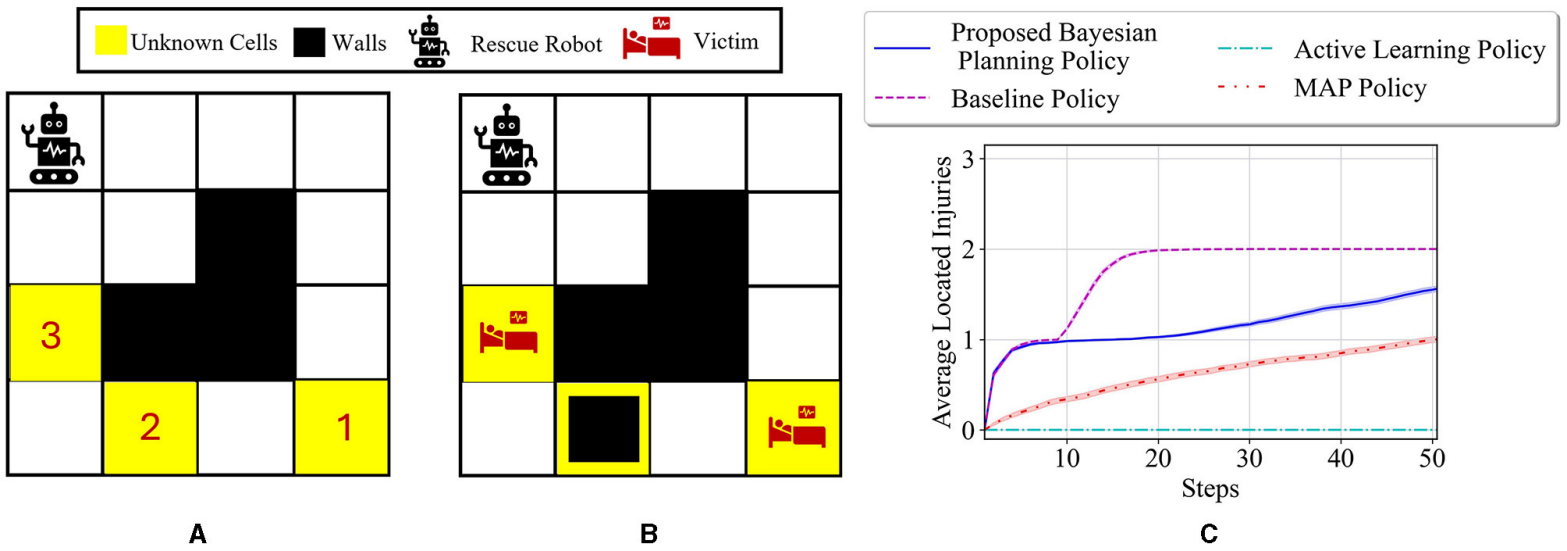
**(A)** Visualization of the 4 × 4 maze problem with three unknown cells. **(B)** The true test environment θ^*^ = [*I, W, I*] with the initial probabilities of unknown cells as $p_{0}^{i}=\left[\frac{1}{3},\frac{1}{3},\frac{1}{3}\right]$ for *i* = 1, 2, 3. **(C)** Performance comparison of different policies for locating the injuries in the test environment.

In this part, our policy is tested for locating injuries in a new 4 × 4 grid which is a smaller grid than the previous 6 × 4 maze problem. This grid, visualized in Figure 8A, has three unknown cells, leading to 27 possible environment models. The state space of this maze can be written as $S=\left\{{\text{s}}^{1},...,{\text{s}}^{13}\right\}$. Considering three auxiliary variables η_*k*_ = {η_1_, η_2_, η_3_} for tracking multiple targets, the belief space size in this problem can be represented as $B=\left\{\left\{1,...,13\right\}\times {\left\{0,1\right\}}^{3}\times \Delta_{27}\right\}$. For training our proposed policy, we consider a case where the priors for the unknown cells are as follows: $p_{0}^{i}=\left[\frac{1}{3},\frac{1}{3},\frac{1}{3}\right]$ for *i* = 1, 2, 3. Furthermore, for testing different policies, a true underlying environment with two injuries and one wall is chosen as represented in Figure 8B. Figure 8C shows the average located injuries achieved by different policies. It is shown that our method has the best performance in all the steps and exhibits a more similar behavior to the baseline policy compared to active learning and MAP policies.

#### 5.1.3 6 × 6 maze problem

**Figure 9:**
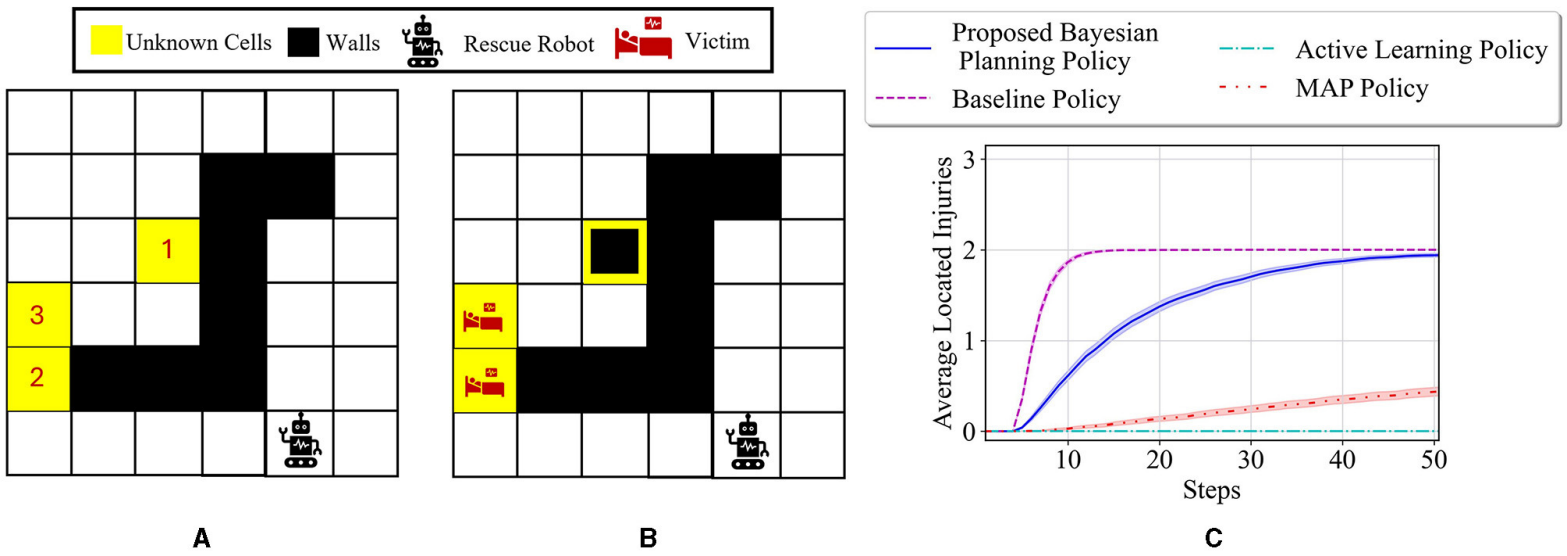
**(A)** Visualization of the 6 × 6 maze problem with three unknown cells. **(B)** The true test environment θ^*^ = [*W, I, I*] with the initial probabilities of unknown cells as $p_{0}^{i}=\left[\frac{1}{3},\frac{1}{3},\frac{1}{3}\right]$ for *i* = 1, 2, 3. **(C)** Performance comparison of different policies for locating the injuries in the test environment.

In this subsection, we study the performance of the proposed method for locating injuries in a 6 × 6 maze with three unknown cells as depicted in Figure 9A, which is a larger maze than the previous two maze problems. This maze has seven walls; this leads to the following state and belief spaces for this maze: $S=\left\{{\text{s}}^{1},...,{\text{s}}^{29}\right\}$, $B=\left\{\left\{1,...,29\right\}\times {\left\{0,1\right\}}^{3}\times \Delta_{27}\right\}$. The prior probabilities of different unknown cells are assumed to be uniform during the training of our policy. Moreover, a maze with two injuries and one wall as shown in Figure 9B is considered for the tests. The performance of our policy, MAP policy, and active learning policy for this experiment is shown in Figure 9C. Our proposed policy reaches a higher value in all the steps in terms of average located injuries compared to the MAP and active learning policies. This figure also denotes that the performance of our method matches the performance of the baseline policy after 50 steps (almost 2), whereas the MAP and active learning policies show a low performance after the same 50 steps (less than 0.5).

### 5.2 Entropy reduction

#### 5.2.1 6 × 4 maze problem

In this part of numerical experiments, we consider the same 6 × 4 maze problem with the objective of quick exploration of an unknown environment. Thus, the reward function in Equation (12) is considered for our experiments, which guides the agent to quickly navigate in the unknown environment and rapidly reduce the overall uncertainty/entropy in the environment models. The state part of the belief can be expressed using 19 potential states for the agent locations without the need for the previously defined auxiliary variables (i.e., used for tracking the injuries). The belief space, in this case, is $B=\left\{\left\{1,...,19\right\}\times \Delta_{27}\right\}$, which is still a large space. Note that in this case, the previously used approaches to compare with our proposed policy cannot be employed. This is due to the fact that the entropy reduction cannot be expressed in terms of the reward for underlying state space. In fact, tracking the posterior probabilities of the environment models is required in this case, which is not possible with the MAP and active learning approaches represented in Equations (21, 22). A one-step entropy reduction policy is employed instead for comparison purposes. This policy selects actions to maximally reduce the next step entropy as


(24) $$\begin{array}{c} \begin{array}{c} {\text{a}}_{k}=\underset{\text{a}\in A}{\mathrm{argmax}}-{𝔼}_{{\text{b}}^{\prime }={\left[{\text{s}}^{\prime },\vartheta^{\prime }\right]}^{T}|\text{b},\text{a}}\left[H\left(\vartheta^{\prime }\right)-H\left(\vartheta \right)\right]. \end{array} \end{array}$$

**Figure 10:**
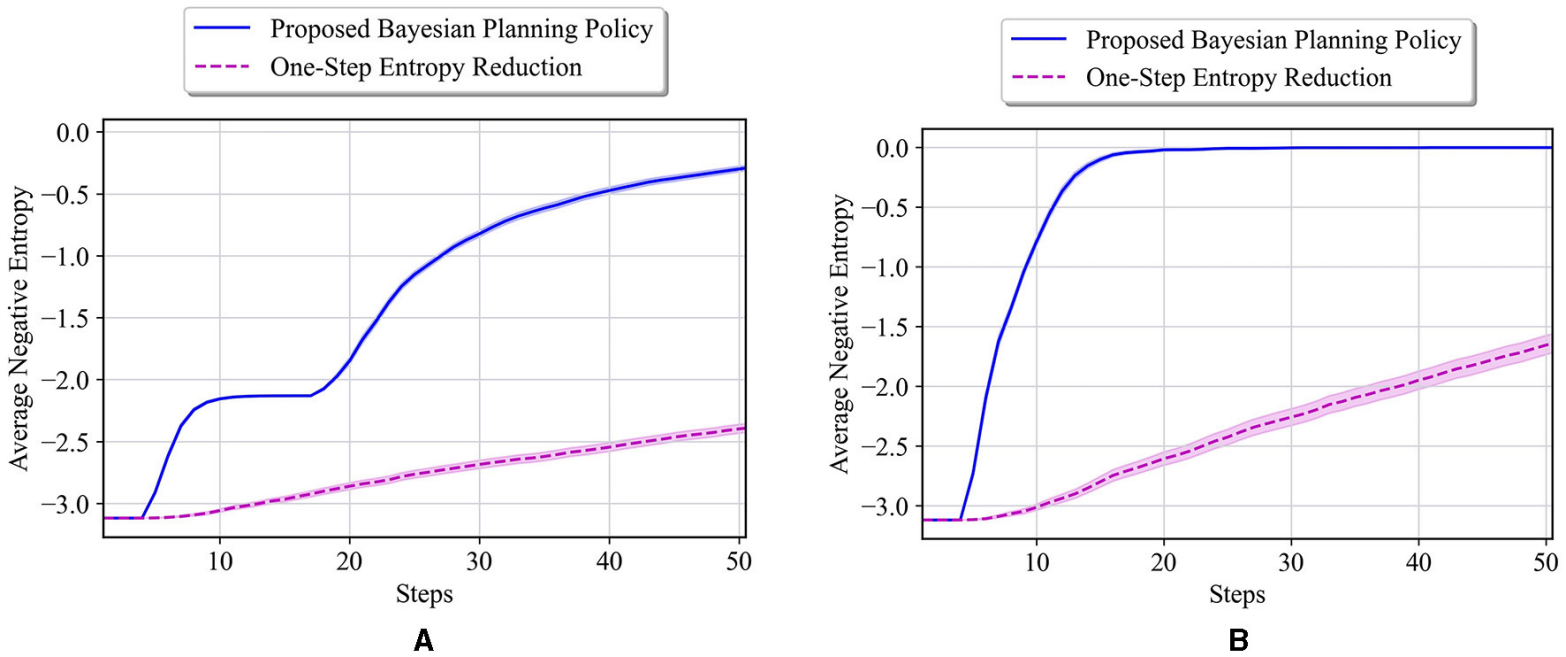
**(A)** Performance comparison in the 6 × 4 maze with the true environment θ^*^ = [*E, E, E*] and initial probabilities of unknown cells as $p_{0}^{i}=\left[\frac{1}{2},\frac{1}{4},\frac{1}{4}\right]$ for *i* = 1, 2, 3. **(B)** Performance comparison in the 6 × 4 maze with the true environment θ^*^ = [*W, W, W*] and initial probabilities of unknown cells as $p_{0}^{i}=\left[\frac{1}{2},\frac{1}{4},\frac{1}{4}\right]$ for *i* = 1, 2, 3.

We consider the following initial probabilities for unknown cells: $p_{0}^{i}=\left[\frac{1}{2},\frac{1}{4},\frac{1}{4}\right]$ for *i* = 1, 2, 3. Based on these priors, the starting value of entropy will be 3.12. Our proposed policy is then tested on two different environments. The first true environment is θ^*^ = [*E, E, E*], where all the unknown cells are empty. The average negative entropy at each step of the test, along with the results of one-step entropy reduction, is shown in Figure 10A. The proposed policy obtains a much faster reduction in the entropy value than the one-step entropy reduction. Moreover, our approach reaches an entropy of 0 (least uncertainty in the environment) in only 20 steps, whereas the one-step entropy reduction shows poor performance until step 50. This demonstrates the importance of accounting for the possible future entropy in making decisions, as opposed to greedy one-step search, which is achieved by the proposed method with policy defined over the belief state. The second test environment consists of all walls in the unknown cells. Figure 10B presents the results of our method and the one-step entropy reduction policy. One can see that the entropy reduction is slower compared to the first test case in Figure 10A, which is due to the fact that all unknown cells are walls and the agent needs much larger time to visit them and reduce the overall uncertainty. However, our proposed approach, again, has achieved a much faster reduction in entropy compared to the one-step entropy reduction policy.

#### 5.2.2 10 × 10 maze problem

**Figure 11:**
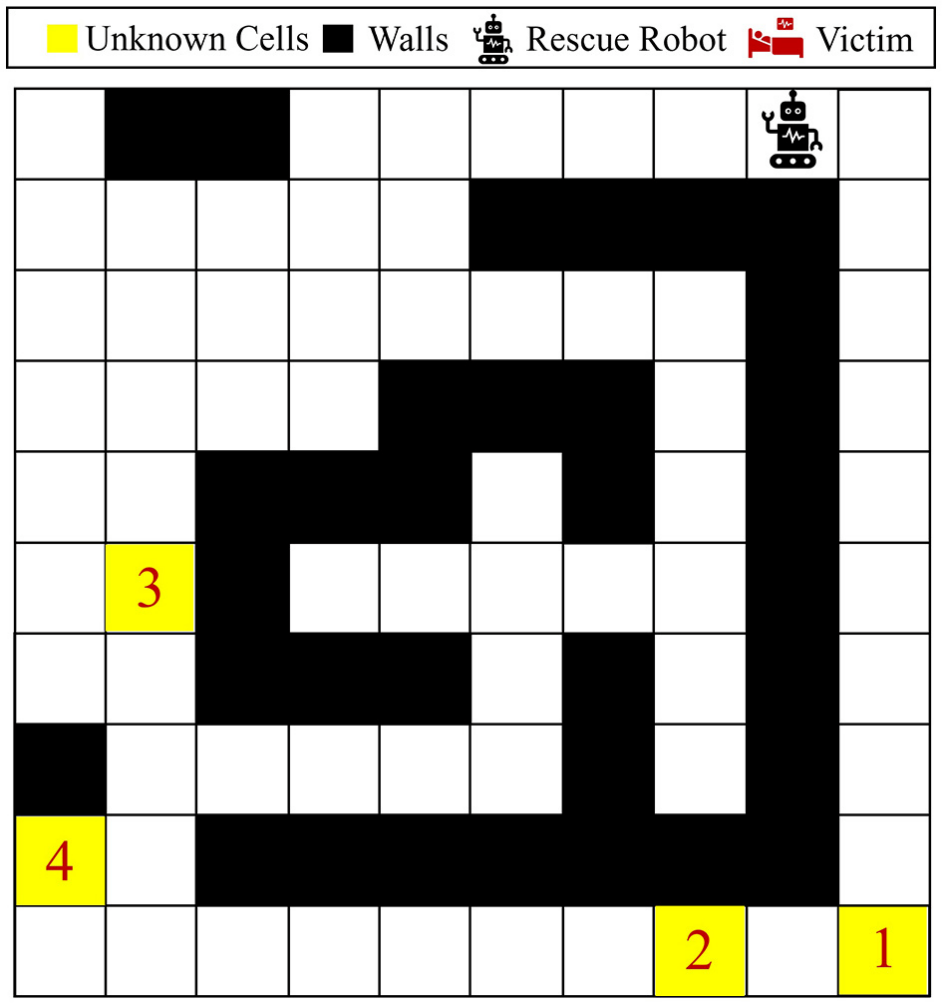
Visualization of the 10 × 10 maze problem. This maze has four unknown cells, where each could be either wall, empty, or victim/injury.

A larger 10 × 10 maze shown in Figure 11 is considered for this part of our numerical experiments. This maze contains four unknown cells, indicated by the color yellow in Figure 11, resulting in 3^4^ = 81 possible maze models. In this part, we aim to tackle the problem of entropy reduction using this larger maze. This maze has 33 walls, so the state space can be represented by $S=\left\{{\text{s}}^{1},...,{\text{s}}^{67}\right\}$. The belief space is $B=\left\{S\times \Delta_{81}\right\}$, which is much larger than the previous maze problems. We consider hyperparameters and maze stochasticity similar to previous problems, except that since this is a larger maze, we consider a horizon of 500 steps for the training process. Finally, 20,000 episodes are used to train our policy in this larger maze.

**Figure 12:**
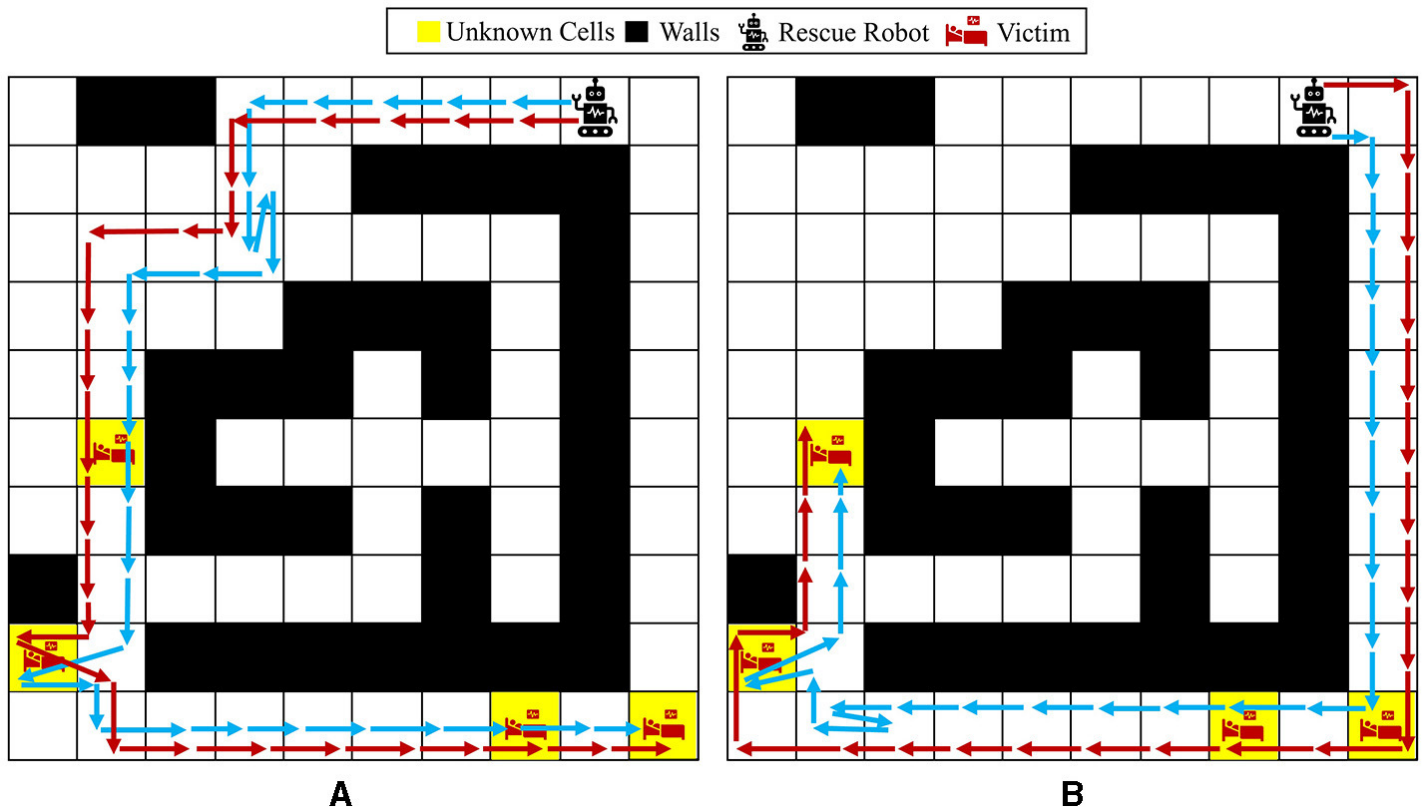
Agent movement trajectories under the proposed Bayesian policy in the 10 × 10 maze; each of the blue and red arrows corresponds to one independent movement trajectory, and the difference in the trajectories is due to the movement stochasticity in the environment. The trajectories are recorded using the proposed policy under two different initial distributions: **(A)** $p_{0}^{1}=\left[\frac{8}{10},\frac{1}{10},\frac{1}{10}\right]$ and $p_{0}^{i}=\left[\frac{1}{12},\frac{6}{12},\frac{5}{12}\right]$ for *i* = 2, 3, 4, **(B)** $p_{0}^{i}=\left[\frac{1}{12},\frac{6}{12},\frac{5}{12}\right]$ for *i* = 1, 2, 3, 4.

The reward function in Equation (12) is used for this part of the experiments, meaning that it is desired for an agent to visit all the unknown cells as quickly as possible and reduce the overall uncertainty in the maze model to 0. Figure 12 represents two independent paths taken by the agent under the proposed Bayesian policy for two different initial probabilities: (a) first unknown cell is set to have a higher chance of being a wall, and the other three unknown cells have a higher chance of being empty or injury, that is, $p_{0}^{1}=\left[\frac{8}{10},\frac{1}{10},\frac{1}{10}\right],p_{0}^{i}=\left[\frac{1}{12},\frac{6}{12},\frac{5}{12}\right]$ for *i* = 2, 3, 4; (b) all the unknown cells have a higher chance of being empty or injury, that is, $p_{0}^{i}=\left[\frac{1}{12},\frac{6}{12},\frac{5}{12}\right]$ for *i* = 1, 2, 3, 4. One can see that in case (a), the agent selects the left path to explore/visit all the unknown cells because unknown cell number 1 has more probability of being a wall and the Bayesian policy predicts a high likelihood that the agent might be stuck in the right side of the maze. However, in case (b), where all the unknown cells have more probability to be an injury or empty, the agent decides to choose the shortest path to get to all the unknown cells, which means that at first it goes from the right side of the maze. This again demonstrates the capability of the proposed method in accounting for potential uncertainty arising from agent movement and model uncertainty for making decisions.

**Figure 13:**
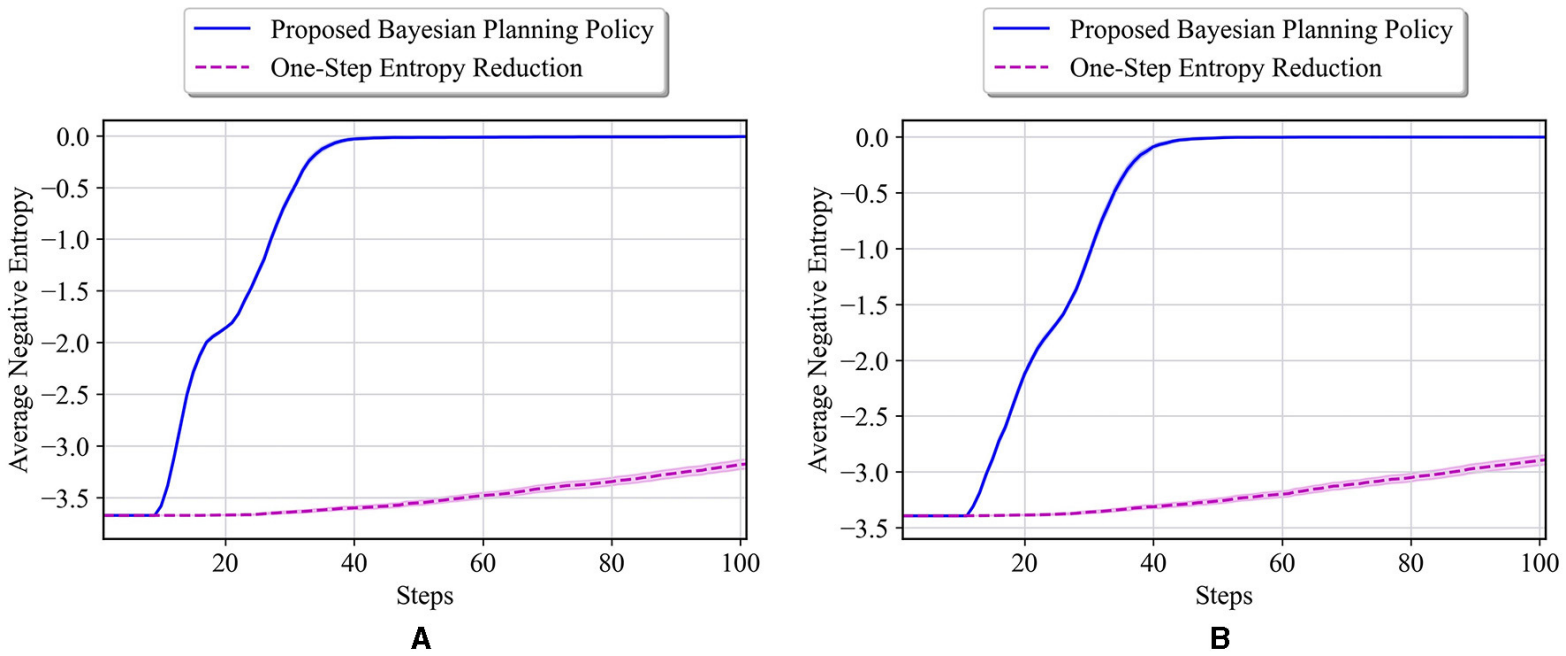
**(A)** Performance comparison in the 10 × 10 maze with the true environment θ^*^ = [*I, I, I, I*] and initial probabilities of unknown cells as $p_{0}^{i}=\left[\frac{1}{12},\frac{6}{12},\frac{5}{12}\right]$ for *i* = 1, 2, 3, 4. **(B)** Performance comparison in the 10 × 10 maze with the true environment θ^*^ = [*I, I, I, I*], and initial probabilities of unknown cells as $p_{0}^{1}=\left[\frac{8}{10},\frac{1}{10},\frac{1}{10}\right]$ and $p_{0}^{i}=\left[\frac{1}{12},\frac{6}{12},\frac{5}{12}\right]$ for *i* = 2, 3, 4.

Once again, similar to Figure 12B, consider that the prior probabilities are set equally for all the unknown cells as: $p_{0}^{i}=\left[\frac{1}{12},\frac{6}{12},\frac{5}{12}\right]$ for *i* = 1, 2, 3, 4, which means that each cell has probability of $\frac{1}{12}$ to be a wall. The starting entropy for this case is equal to 3.67. For this case, the average results of our proposed policy and one-step entropy reduction policy for the true environment θ^*^ = [*I, I, I, I*] are shown in Figure 13A. Our proposed policy outperforms the one-step entropy reduction results by a far margin in all the steps, and it reaches an entropy of zero after only 40 steps. As a final scenario, only the prior probability of being a wall for the first unknown cell is set to be larger as opposed to the previous case, that is, $p_{0}^{1}=\left[\frac{8}{10},\frac{1}{10},\frac{1}{10}\right]$ (similar to Figure 12A). The starting entropy value, in this case, is 3.39, and the test results on θ^*^ = [*I, I, I, I*] are presented in Figure 13B. As seen in this figure, our policy has a better performance in all the steps compared to one-step entropy reduction. Our navigation policy results in zero entropy after about 45 steps, where the one-step entropy reduction fails to achieve good performance over all 100 steps.

## 6 Conclusion and future works

This study developed a reinforcement learning Bayesian planning policy for rescue operations in unknown or partially known environments. Unlike most existing approaches that rely on the availability of an exact model or simulator for representing the underlying environment, this study considers realistic problems in which no or limited prior information about the environment might be available. A new Bayesian formulation of navigation in unknown and uncertain environments is provided using the definition of belief state, which tracks the agent state and the uncertainty of the environment up to each step. This formulation is used to formulate the optimal Bayesian policy, which can be computed through the propagation of all the uncertainty in the agent state and environment models. A solution to the optimal Bayesian policy is introduced using a deep reinforcement learning method. Finally, the performance of the proposed method is demonstrated using different maze problems with various uncertainties. Note that the proposed method is versatile and can be applied not only to environments with discrete state spaces but also to those with continuous state spaces or large discrete state spaces.

The computational complexity of the proposed method increases exponentially with the number of unknown cells in the environments, potentially limiting its scalability. Our future work includes studying the scalability of the proposed policies in domains with extremely large belief spaces and different uncertainties in the model. We will also extend the idea of Bayesian planning to domains with partially observable states, as well as domains with multiple agents and continuous action spaces.

## Data availability statement

The raw data supporting the conclusions of this article will be made available by the authors, without undue reservation.

## Author contributions

MA: Conceptualization, Data curation, Formal analysis, Investigation, Methodology, Software, Validation, Visualization, Writing – original draft, Writing – review & editing. MI: Formal analysis, Funding acquisition, Methodology, Project administration, Resources, Supervision, Validation, Writing – original draft, Writing – review & editing, Conceptualization, Investigation.

## Appendix

In this section, we provide the run times of our proposed method and the compared approaches for selected experiments to highlight the limitations and strengths of our method. Table A1 showcases the run times of the experiments using different methods. The reported times were recorded using a system with two 2.4 GHz Intel E5-2680 v4 CPUs and 64 GB RAM memory. As can be seen in Table A1, the run times of different approaches are divided into training times and test/execution times. The training time for our method refers to the time needed for training the weights of the neural network, and the training time for active learning and MAP refers to the time needed for training the policies for each of the possible environments in each experiment. Note that active learning and MAP use the same trained policies during the execution, therefore they have similar training times. The test/execution time is the time that was used to record the results over 1,000 trials for different methods. From Table A1, one can see that our method is much faster in terms of training compared to active learning and MAP. The execution time for different methods is however in the same range, with our method being faster overall throughout all the experiments. Further, it can be seen that the complexity of the problem increases as the number of unknown cells (Table 1, Four Unknown Cells) and the grid size (Figure 9) increases, and this naturally results in longer training and test/execution times as demonstrated in Table A1.

**Table A1:** Training and execution times of different policies in selected experiments.

	**Training time (minutes)**			**Test/execution time (minutes)**		
	**Our method**	**Active learning**	**MAP**	**Our method**	**Active learning**	**MAP**
Figure 4A	126	1,247	1,247	11	20	17
Figure 4B	98	1,118	1,118	9	19	16
Figure 5	153	1,331	1,331	12	21	17
Figure 6	158	1,342	1,342	12	23	18
Table 1–two unknown cells	97	1,015	1,015	7	16	14
Table 1–three unknown cells	148	1,301	1,301	11	19	17
Table 1–four unknown cells	169	1,375	1,375	13	21	18
Figure 8	82	949	949	7	15	11
Figure 9	194	1,417	1,417	16	26	22
